# Retailer Stackelberg game in a supply chain with pricing and service decisions and simple price discount contract

**DOI:** 10.1371/journal.pone.0195109

**Published:** 2018-04-12

**Authors:** Seyed Jafar Sadjadi, Hashem Asadi, Ramin Sadeghian, Hadi Sahebi

**Affiliations:** 1 Department of Industrial Engineering, Iran University of Science and Technology, Tehran, Tehran, Iran; 2 Industrial Engineering Group, Iran University of Payame Noor, Farmanieh, Tehran, Iran; Southwest University, CHINA

## Abstract

This paper studies the Retailer Stackelberg game in a supply chain consisting of two manufacturers and one retailer where they compete simultaneously under three factors including price, service and simple price discount contract. It is assumed that the second manufacturer provides service directly to his customers, and the retailer provides service for the first product’s customers, while the retailer buys the first product under price discount from the first manufacturer. The analysis of the optimal equilibrium solutions and the results of the numerical examples show that if a manufacturer chooses the appropriate range of discount rate, he will gain more profit than when there is no discount given to the retailer. This situation can be considered as an effective tool for the coordination of the first manufacturer and the retailer to offer discount by manufacturer and to provide the service by the retailer. We obtain equilibrium solution of Retailer Stackelberg game and analyze the numerical examples under two cases: **a)** the manufacturers sell their products to the retailer without price discount contract. **b)** The first manufacturer sells his products to the retailer with the simple price discount contract. The preliminary results show that the service and the price discount contract can improve the performance of supply chain.

## 1. Introduction

When the intensity of competition in the business environment increases, many factors affect the profit of supply chain members. Some examples of these factors include services, different types of discount, delivery time, warranty, advertising, quality, etc. Nowadays, many manufacturers such as IBM, HP, Dell, Nike and Apple and many retailers such as Walmart and Target are successful because of offering support and after-sales services to customers.

Researches show that providing after-sale or pre-sale services will increase the demand of product and the company's profit. Therefore, in today's complex competitive environment, companies or manufacturers should focus on services and provide various services to attract customers or to use some encouraging policies to consign their services to the retailers. In this research, one primary question is whether the price discount contract can be considered as an incentive policy for retailers to provide appropriate service for customers. Therefore, it is important to examine the effects of services and price discount contracts on the demand and profit of the supply chain members.

There are many existing studies on the competition in supply chain, but we will concentrate on those which are related to the competition in supply chain under price, service level and simple price discount contract. This subject has been reviewed in many papers, however, there is not any study that examines these factors in competition in the supply chain simultaneously. Thus, we examine this topic in the supply chain.

Most of the studies have been conducted on only one of the fields of service provision by the retailer or directly by the manufacturer, but the provision of syndicated services by both the retailer and the manufacturer with our paper’s supply chain structure has been less studied. A sample competition under this structure can be seen in the area of software support services after hardware sales.

For example, Wal-Mart retailer sells some of the HP's laptops to the customers with software packages, mouse or bag, but the Hewlett-Packard Company does not provide such services to customers in its online sales. The Acer Company sells some of laptops with office 365 personal or Microsoft office trial.

One of the actions to improve the performance of supply chains is the coordination among members. The aim of our research is to examine the effects of service and price discount contract on the profit, the demand and coordination of supply chain’s members. Our other questions in this research include:

a) How does providing services for customers influence on increasing the profit and demand of supply chain members?
b) Does offering price discount by manufacturer to retailer provide an appropriate tool to improve the performance of supply chain members?
c) Does offering price discount by manufacturer to retailer provide a coordination tool among them?
d) Does the increasing the discount rate always increase or permanently reduce the manufacturer's demand and profit?

The remainder of this paper is organized as follows. The next section reviews the literature related to competition in supply chain under price, service and price discount adopting game-theoretic approach. Section 3 describes the model description and assumptions. In Section 4, the numerical examples and analysis are presented. The conclusion is given in the last section. The detailed proofs of the propositions presented are included in the S1 Appendix.

## 2. Literature review

### 2.1. Service level

For the proposed main model of this paper, the services are actions where the manufacturer or the retailer performs in order to attract customers to buy and persuade them to pay more for the product. Examples of services include post-sale customer support, on-time product delivery, responsive product repair, field trials, professional shopping advice and guarantees, etc. This definition of service was used in all the existing literature review. There are many studies in which the manufacturers or the retailers or both of them provide services for the customers, and we review a large number of them.

Ali et al. [1] showed that the demand disruptions in the retail markets could significantly influence on price and service levels. By modeling two Stackelberg games, Yu and Xiao [2] investigated the effects of channel leadership on the service and price decision and profit in a supply chain including one supplier, one retailer and one third-party logistics provider. In a system consisting of two remanufacturers and a common retailer with uncertain demand and condition of the acquired items, Jena and Sarmah [3] examined competition under price and service. Wang et al. [4] modeled four games in a dual supply chain where retailer provides service for both products and compared the effectiveness of the optimal results by using some numerical example results.

Li and Bo Li [5] showed that increasing customer loyalty to the retail channel leads to channel efficiency growth and increasing the retailer’s fairness concerns leads to channel efficiency fall. Zhang et al. [6] analyzed the impacts of retail services and the degree of customer loyalty to the retail channel in a dual-channel closed-loop supply chain with the remanufactured product and the new product. Chen et al. [7] examined the impact of power various structures on the retail service in a supply chain including of offline and online channels. By using the numerical examples, Wang and Zhao [8] showed the effects of the degree of customer loyalty to the retail channel on optimal service levels. Dan et al. [9] explored the influence of retailers’ power in a dual channel supply chain consisting of a manufacturer and a common retailer and an electronic retailer. Chen and Yang [10] examined service cooperation in a dual-channel supply chain where the manufacturer consign his service to the retailer. Li et al. [11] investigated the effects of production cost disruptions and demand on optimal pricing, service level, and production decisions. Pei and Yan [12] showed that the retail services could be used as an incentive to coordinate the relationship between members.

Kurata and Nam [13] modeled five scenarios to examine the effect of information structures uncertainty on after-sales service. In a supply chain including one supplier, e-commerce channel and a common retailer that provides retail services, Lu and Liu [14] modeled a Nash pricing game and two types of Stackelberg pricing games. Chen et al. [15] presented a supply chain including a manufacturer with retail and Internet channels and the other manufacturer only with the retail channel. They showed that an increased service level may reduce the Internet channel threat for the retailer and increase the manufacturer’s profit. The uncertain demand influences on the rm’s optimal retail service and profit [16]. In a centralized and a decentralized dual-channel supply chain, Dan et al. [17] examined the impacts of retailer service on the manufacturer and the retailer’s pricing decisions.

Wu [18] considered a supply chain consisting of two manufacturers in which the first manufacturer produces the new product and the second manufacturer produces the remanufactured product and a common retailer. In this supply chain, they investigated competition under service and price. Lu et al. [19] analyzed a vertical Nash and a manufacturer Stackelberg and a retailer Stackelberg game in a supply chain with two manufacturers that provide services directly to customer and showed that the consumers receive higher service level in Vertical Nash game.

Kurata and Nam [20] examined the role of after-sales service including optional services in a supply chain. In a dual channel supply chain, the manufacturer uses the direct channel as a motivation tool of providing the improved retail services [21]. In a supply chain including a supplier and two retailers with retail services to customer, Yao et al. [22] obtained the condition under which two retailers are reluctant to share their information with the supplier. Xia and Gilbert [23] studied the impact of the demand enhancing services on the strategic interactions between the manufacturer and the dealer.

Tsay and Agrawal [24] studied a distribution system in which a manufacturer supplies a common product for two independent retailers, who in turn uses service as well as retail price to directly compete for end customers. Xu et al. [25] examined the effect of fairness concerns on the retailer’s service and revenue-sharing decisions by presenting three different scenarios. In a fuzzy uncertainty environment, Zhao and Wang [26] investigated the pricing and retail service policies. Lu et al. [27] explored the effects of repeated transactions by consumers and manufacturers on optimal decisions of members such as price, service level and order quantity by considering multiple periods. Bin Wang and Jing Wang [28] investigated three scenarios including of Nash Equilibrium, Enterprise Alliance and Stackelberg in a supply chain with substitutable goods and providing service.

In a dual channel supply chain in which the choice of the customer purchase channel depends on price and service qualities, Dumrongsiri et al. [29] showed that an increase in retailer’s service quality may increase the manufacturer’s profit and a wide range of customer that service is important for them may benefit both factions in the dual channel. Tsay and Agrawal [30] presented a dual channel supply chain consisting of a manufacturer and a reseller to investigate different ways to regulate the manufacturer and reseller relationship. Gerhard Aust [31] presented a model to investigate the competition under price, product quality and retail service’s factors in a three-echelon supply chain.

### 2.2. Price discount

Many researchers examined the competition in supply chain under discount factor, but a few of them are similar to the proposed model of this paper. We use simple price discount contract in supply chain similar to [32–33].

Gangshu et al. [32] examined the effects of different price discount contracts and pricing schemes on supply chain. They showed that the simple price discount contracts could improve the whole supply chain’s performance. Bernstein and Federgruen [33] coordinated the supply chain by using a linear price-discount sharing scheme.

Many papers studied competition in supply chain under another type of discount that are not similar to this paper, for example, Nie and Du [34] examined quantity discount contracts in a supply chain consisting of a manufacturer and a retailer. Zissis et al. [35] obtained closed form expressions of the quantity discounts that minimize the manufacturer's costs. In a supply chain including of a supplier, a retailer, and a carrier, Li et al. [36] obtained wholesale-price discount scheme to coordinate the supply chain by using the profit sharing method. In an automobile supply chain, Luo et al. [37] showed that the discount rate and the subsidy ceiling together lead to the effective incentive scheme.

Khouja et al. [38] used the gift cards as a discount scheme to consumers. In a supply chain consist of a manufacturer and a retailer, Chen [39] coordinated the supply chain with a wholesale-price-discount scheme. Yue et al. [40] investigated the coordination of cooperative advertisement between members when the manufacturer offers price deductions to customers. In this study, the wholesale price is a discounted rate of the retail price and the simple price discount contract is similar to the definition used by [32–33].

Xie et al. [41] presented a wholesale price-discount scheme that can induce the retailers to voluntarily participate in early order commitment. In a decentralized supply chain, by using of the quantity discounts in a game theoretic model, Barbara and Ventura [42] examined the coordination between a supplier and a buyer.

In a three-level supply chain with stochastic demand, Bai and Wang [43] explored the price discount contract coordination and obtained the optimal profits. By using the lot size based discount, Routroy et al. [44] presented a mathematical model for a supply chain coordination. In a distribution system, Hsieh et al. [45] used the price discount as a mechanism for the coordination between the distributor and the retailer.

**Table 1** illustrates the comparison of our paper to other relevant papers. This comparison shows the similarities and differences of the proposed model with other relevant papers. However, in none of the studies mentioned in Table 1, pricing, service and price discount decisions was considered, simultaneously. Also, in the supply chain of the proposed model of this paper, the structure of service provision and the bargaining power are different for the majority of relevant papers.

**Table 1:** Summary table of the existing literature.

Structure of supply chain	Service provider	Decisions structure	Type of discount	Reference
1 Manufacturer, 1 Retailer	Manufacturer	Pricing, service and production	-	Li et al. [11]
2 Manufacturers, 1 Retailer	2 Manufacturers	Pricing and service	-	Wu [18]
2 Manufacturers, 1 Retailer	2 Manufacturers	Pricing and service	-	Lu et al. [27]
2 Manufacturers, 1 Retailer	2 Manufacturers	Pricing and service	-	Wang and Jing Wang [28]
2 Manufacturers, 1 Retailer	2 Manufacturers	Pricing and service	-	Jena and Sarmah [3]
1 Manufacturer 1 Physical Retailer E-commerce (e-tailers) channels	Physical retailer	Pricing and service	-	Lu and Liu [14]
1 Manufacturer 1 Independent retailer 1 Internet channel	Independent retailer	Pricing, service, fairness concern revenue sharing	-	Xu et al. [25]
2 Manufacturers 1 Traditional retailer 1 Internet channel	Traditional retailer	Pricing and service	-	Chen et al. [15]
1 Manufacturer 1 Traditional retailer 1 Online direct channel	Traditional retailer	Pricing and service	-	Bin Dan et al. [17]
1 Manufacturer 1 Traditional retailer 1 Electronic channel (e- retailer)	Traditional retailer	Pricing and service	-	Dan et al. [9]
1 Manufacturer 1 Traditional retailer 1 Online direct channel	Traditional retailer	Pricing and service	-	Chen and Yang [10]
1 Manufacturer 1 Traditional retailer 1 Internet channel	Traditional retailer	Pricing and service	-	Zhang et al. [6]
1 Manufacturer 1 Traditional retailer 1 Online direct channel	Traditional retailer	Pricing and service	-	Pei and Yan [12]
2 Manufacturers 1 Traditional retailer 1 Internet channel	Traditional retailer	Pricing and service	-	Wang et al. [4]
1 Manufacturer 1 Traditional retailer 1 Online direct channel	Traditional retailer	Pricing, service and fairness concern	-	Li and Bo Li [5]
1 Supplier, 1 Retailer 1 Online direct channel	Retailer	Pricing and service	-	Chen et al. [7]
1 Manufacturer, 2 Retailers	2 Retailers	Pricing and service	-	Yao et al. [22]
1 Manufacturer, 2 Retailers	2 Retailers	Pricing and service	-	Tsay and Agrawal [24]
1 Manufacturer, N Retailers	N Retailers		-	Ali et al. [1]
1 Manufacturer 1 Traditional retailer 1 Online direct channel	Manufacturer and Retailer	Pricing and service	-	Wang and Zhao [8]
1 Manufacturer 1 Retailer	Manufacturer and Retailer	Service	-	Kurata and Nam [13]
1 Manufacturer 1 Retailer	Manufacturer and Retailer	Service	-	Kurata and Nam [20]
1 Manufacturer 1 Dealer	Manufacturer and Dealer	Pricing and service	-	Xia and Gilbert [23]
1 Manufacturer 1 Traditional retailer 1 Online direct channel	Manufacturer and Retailer	Pricing, service and order quantity	-	Dumrongsiri et al.[29]
1 Manufacturer 1 Traditional retailer 1 Online direct channel	Manufacturer and Retailer	Pricing and service	-	Tsay and Agrawal [30]
1 Supplier, 1 Retailer 1 Online direct channel	-	Pricing	Price discount contracts	Gangshu et al. [32]
1 Supplier N Retailers	-	Pricing and inventory	Price discount contracts	Bernstein and Federgruen [33]
1 Manufacturer 2 Retailers	-	Pricing and fairness concern	Quantity discount	Nie and Du [34]
1 Supplier, 1 Retailer 1 Carrier	-	Pricing and order quantity	Wholesale-price discount	Li et al. [11]
1 Manufacturer 1 Retailer	-	Pricing	Price discount and subsidy	Luo et al. [37]
N suppliers, 1 Retailer	-	Order quantity	Free gift cards	Khouja et al. [38]
1 Manufacturer 1 Retailer	-	Order quantity	Wholesale-price-discount	Chen [39]
1 Manufacturer 1 Retailer	-	Advertising and pricing	Price deductions	Yue et al. [40]
1 Supplier 1 Buyer	-	Pricing and inventory	Quantity discounts	Barbara and Ventura [42]
1 Manufacturer N Retailers	-	Order quantity	Wholesale price-discount	Xie et al. [41]
1 Supplier 1 Retailer	-	Production and inventory	Wholesale-price contract	Dong and Zhu [46]
1 Supplier, 1 Manufacturer 1 Retailer	-	Order quantity and pricing	Price discount contract	Bai and Wang [47]
1 Manufacturer 3 Retailers	-	Order quantity	Lot size based discount	Routroy et al. [48]
1 Distributor N Retailers	-	Order quantity and pricing	Price discount	Hsieh et al. [45]
***2 Manufacturers*** ***1 Retailer***	***Retailer and one of the manufacturers***	***Pricing*, *service and order quantity***	***Price discount contracts***	*Our paper

## 3. Main model

**Fig 1:**
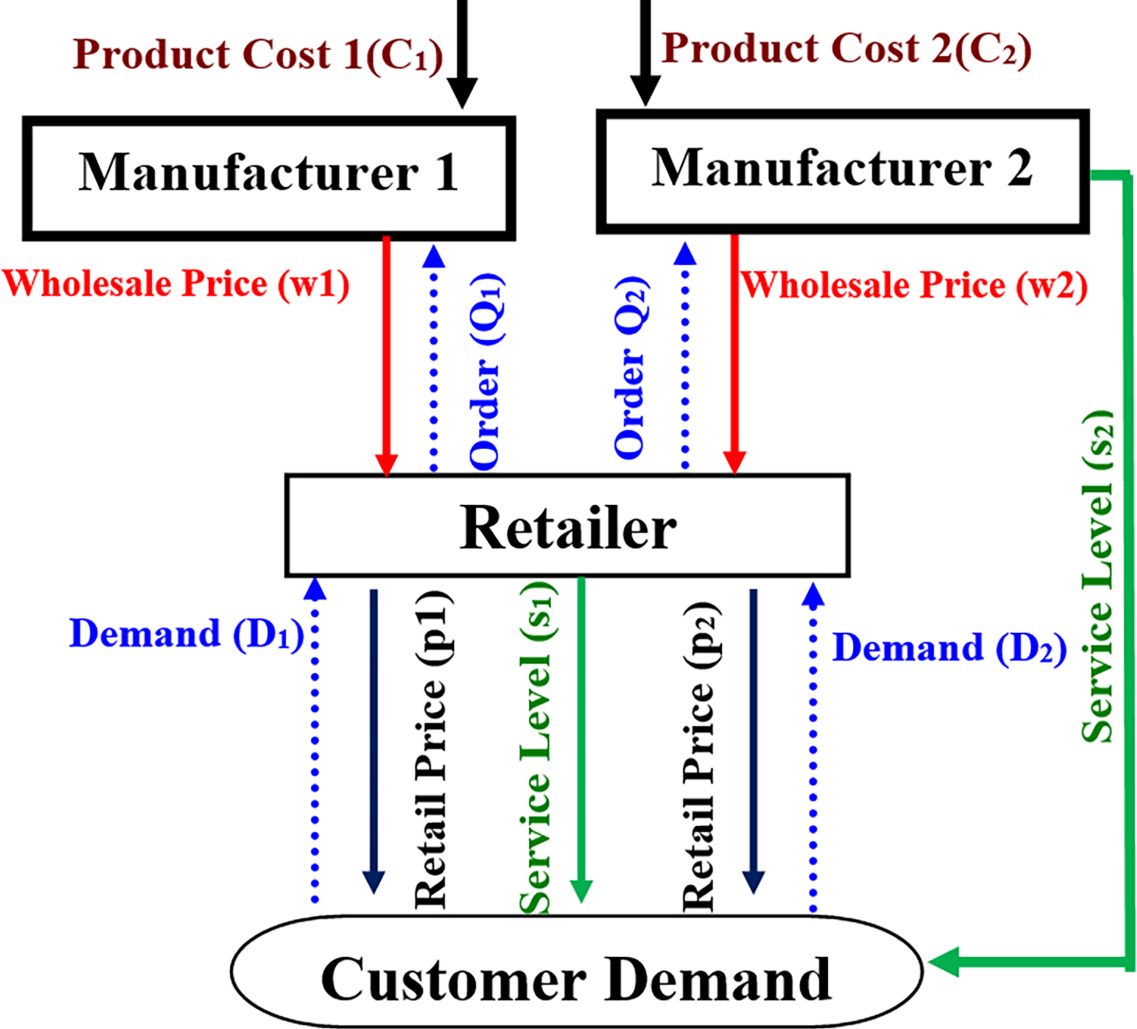
Supply chain structure in first case.

The supply chain of this paper consists of two manufacturers and a common retailer. The manufacturers sell their products to the end consumers by the retailer (Fig 1). In this model, all the members of supply chain try to maximize their own profits and there is not any cooperation among the members. Each member of the supply chain has perfect information about the other members. It is assumed that the two manufacturers have equal bargaining power and the retailer has more bargaining power than the manufacturers. Therefore, the retailer is market's leader.

**Fig 2:**
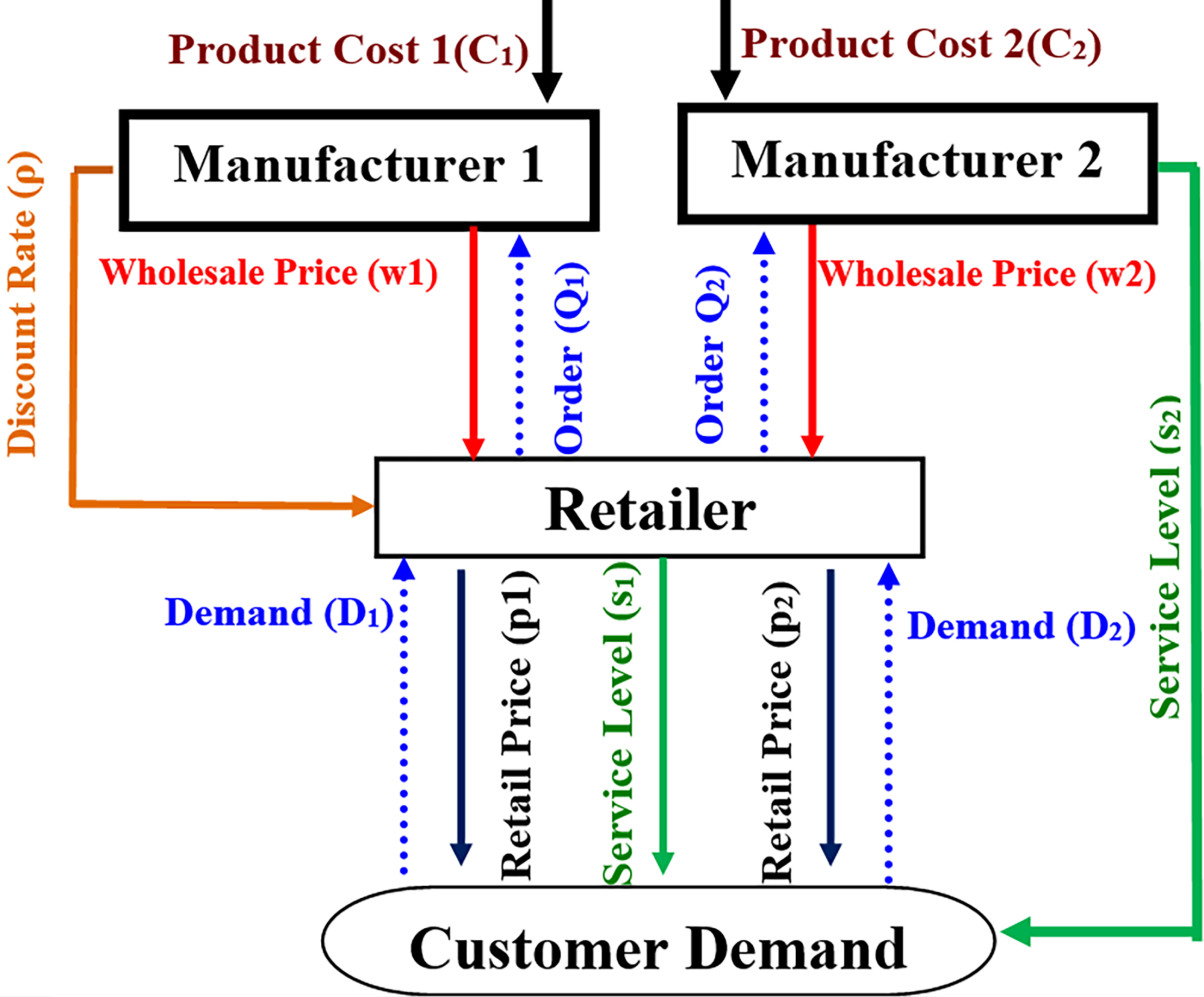
Supply chain structure in second case.

The demands for the products of two manufacturer depend on the retail prices and the service levels and they are considered to be deterministic. The retailer sells both products to the customers and provides services to the first product’s customers and the second manufacturer provides services directly to his customers. We analyze two Retailer Stackelberg models under two single period cases including:

a) The Manufacturers sell their products to the retailer without price discount (Fig 1).
b) The first manufacturer sells his products to the retailer with simple price discount and the second manufacturer does not provide any price discount (Fig 2).

### 3.1. First case: Without simple price discount contract

We consider demand *Q*_*i*_ that is similar to the demand function used in the literature [18–24–47]. The following notations in Table 2 are used. We define the general demand function for product *i* that captures product and service competition as follows:

$$Q_{i}(p_{1},p_{2},s_{1},s_{2})=a_{i}-b_{p}p_{i}+\theta_{p}(p_{j}-p_{i})+b_{s}s_{i}-\theta_{s}(s_{j}-s_{i})$$ (1)

where *a*_*i*_ > 0, *b*_*p*_ > 0, *b*_*s*_ > 0, *θ*_*p*_ > 0, *θ*_*s*_ > 0, and *i*,*jϵ*{1,2} *i* ≠ *j*.

**Table 2:** Notations of parameters and variables.

Symbol	Description
*D*_*i*_	Market demand of product *i iϵ{*1,2}
*Q*_*i*_	Quantity of products ordered by retailer from manufacturer *i*
*a*_*i*_	The market base of product *i*
*b*_*p*_	Price elasticity on market demand
*b*_*s*_	Service elasticity on market demand
*θ*_*p*_	Intensity of price competition
*θ*_*s*_	Intensity of service competition
*η*_1_	Service cost coefficient of retailer
*η*_2_	Service cost coefficient of manufacturer 2
*c*_*i*_	Manufacturer *i*’s product cost
*w*_*i*_	Wholesale price of product *i*
*p*_*i*_	Retailer price of product *i*
*s*_1_	Service level provided by retailer
*s*_2_	Service level provided by manufacturer 2
*π*_*Mi*_	Profit function of manufacturer *i*
*π*_*R*_	Profit function of retailer

The first manufacturer carries the production cost and the second manufacturer carries the production cost and service cost. The cost of providing *s*_2_units of service by the second manufacturer is $\frac{\eta_{2}s_{2}^{2}}{2}$, where *η*_2_ is the ultimate cost of service and the cost of providing *s*_1_ units of service by the retailer is $\frac{\eta_{1}s_{1}^{2}}{2}$, where *η*_1_ is the service cost coefficient of the retailer as is given in the literature [12–14–17–18–19–22–24–46–47–48]. Therefore, the profit functions for two manufacturers are:

$$\pi_{M1}=(w_{1}-c_{1})Q_{1},$$ (2)

$$\pi_{M2}=(w_{2}-c_{2})Q_{2}-\frac{\eta_{2}s_{2}^{2}}{2}$$ (3)

The costs to the retailer include wholesale prices and retail services. Therefore, the retailer’s profit function is given as follows,

$$\pi_{R}=(p_{1}-w_{1})Q_{1}-\frac{\eta_{1}s_{1}^{2}}{2}+(p_{2}-w_{2})Q_{2}$$ (4)

#### 3.1.1. Retailer Stackelberg

The Retailer Stackelberg game occurs in markets where the size of the retailers is large compared with their suppliers or manufacturers. For example, the size of the retailers such as Walmart is large compared with their suppliers and they can infiuence the sales of each product by lowering price and they are leader in the market. In a Stackelberg game, according to the follower's response function, the leader makes a decision to maximize his own profit. First, the optimal reaction functions for the two manufacturers are obtained, given that the manufacturers have observed the decisions made by the retailer. Then, the retailer’s equilibrium solutions are obtained when he knows how the manufacturers would react to his decisions.

Manufacturers Reaction Functions: The first manufacturer must choose wholesale price $w_{1}^{*}$ and the second manufacturer must choose wholesale price $w_{2}^{*}$ and service level $s_{2}^{*}$ to maximize their equilibrium profit. Then, the reaction functions of the manufactures are:

$$w_{i}^{*}\in \arg\;\max_{w_{i}}\;\pi_{M_{i}}(w_{i},w_{j}^{*},s_{2}^{*}|p_{1},p_{2},s_{1})$$ (5)

$$s_{2}^{*}\in \arg\;\max_{s_{2}}\pi_{M_{2}}\;(w_{1}^{*},w_{2}^{*},s_{2}|p_{1},p_{2},s_{1})$$ (6)

where $\pi_{M_{i}}(w_{1},w_{2},s_{2}|p_{1},p_{2},s_{1})$ given by Eq (2) and Eq (3) denote the profit to the manufacturers at this stage when they set the wholesale prices *w*_1_, *w*_2_ and the second manufacturer sets service level *s*_2_, given earlier decisions on retail price and service level *p*_1_, *p*_2_ *and s*_1_ by the retailer. The Proposition 1 gives the results.

Proposition 1. In the Retailer Stackelberg game, for a given retail prices *p*_1_, *p*_2_ and *s*_1_, the manufacturers reaction functions are obtained as:

$$w_{1}^{*}=I_{2}-G_{2}p_{1}-H_{2}p_{2}-K_{2}s_{1},$$ (7)

$$w_{2}^{*}=J_{2}-M_{2}p_{1}-L_{2}p_{2}-N_{2}s_{1}$$ (8)

$$s_{2}^{*}=O_{2}-V_{2}p_{1}-U_{2}p_{2}-Y_{2}s_{1}$$ (9)

where *I*_2_, *G*_2_, *H*_2_, *K*_2_, *J*_2_, *M*_2_, *L*_2_, *N*_2_, *O*_2_, *V*_2_, *U*_2_ *and Y*_2_ are defined in S1 Appendix. The proof of Proposition 1 is given in S1 Appendix.

Retailer Decision: By using the reaction functions of manufacturers, we can obtain the retailer's optimal retail prices and services. The retailer’s best response functions for retail prices *p*_1_, *p*_2_ and service level *s*_1_ are obtained by maximizing retailer's profit, given $w_{i}^{*}$ and $s_{2}^{*}$ in Eq (7), Eq (8) and Eq (9), respectively. This leads to

$$p_{i}^{*}\in \arg\;\max_{p_{i}}\pi_{R}\;(p_{i},p_{j}^{*},s_{1}^{*})$$ (10)

$$s_{1}^{*}\in \arg\;\max_{s_{1}}\pi_{R}\;(p_{i},p_{j}^{*},s_{1}^{*})$$ (11)

The Proposition 2 gives the results.

Proposition 2. In the Retailer Stackelberg game, the retailer’s optimal retail prices and the optimal retail service level, denoted as $p_{1}^{*},\;p_{2}^{*}$ and $s_{1}^{*}$ are given as follows:

$$p_{1}^{*}=\frac{\sigma_{2}\tau_{2}-\xi_{2}\varepsilon_{2}}{\delta_{2}\sigma_{2}-\xi_{2}\vartheta_{2}}$$ (12)

$$p_{2}^{*}=\frac{\delta_{2}\varepsilon_{2}-\vartheta_{2}\tau_{2}}{\delta_{2}\sigma_{2}-\xi_{2}\vartheta_{2}}$$ (13)

$$s_{1}^{*}=\frac{\gamma_{2}\sigma_{2}\tau_{2}-\xi_{2}\varepsilon_{2}\gamma_{2}+\phi_{2}\delta_{2}\varepsilon_{2}-\phi_{2}\vartheta_{2}\tau_{2}+\delta_{2}\sigma_{2}\beta_{2}-\xi_{2}\vartheta_{2}\beta_{2}}{-2\lambda_{2}(\delta_{2}\sigma_{2}-\xi_{2}\vartheta_{2})}$$ (14)

where *σ*_2_, *τ*_2_, *ξ*_2_, *ε*_2_, *δ*_2_ *ϑ*_2_, *γ*_2_, *ϕ*_2_, *β*_2_ and *λ*_2_ are defined in S1 Appendix. The proof of Proposition 2 is given in S1 Appendix.

By substituting Eq (12), Eq (13) and Eq (14) into Eq (7), Eq (8) and Eq (9), the manufactures optimal wholesale prices and the second manufacturer optimal service level are obtained as follows:

$$w_{1}^{*}=I_{2}-G_{2}\frac{\sigma_{2}\tau_{2}-\xi_{2}\varepsilon_{2}}{\delta_{2}\sigma_{2}-\xi_{2}\vartheta_{2}}-H_{2}\frac{\delta_{2}\varepsilon_{2}-\vartheta_{2}\tau_{2}}{\delta_{2}\sigma_{2}-\xi_{2}\vartheta_{2}}-K_{2}\frac{\gamma_{2}\sigma_{2}\tau_{2}-\xi_{2}\varepsilon_{2}\gamma_{2}+\phi_{2}\delta_{2}\varepsilon_{2}-\phi_{2}\vartheta_{2}\tau_{2}+\delta_{2}\sigma_{2}\beta_{2}-\xi_{2}\vartheta_{2}\beta_{2}}{-2\lambda_{2}(\delta_{2}\sigma_{2}-\xi_{2}\vartheta_{2})}$$ (15)

$$w_{2}^{*}=J_{2}-M_{2}\frac{\sigma_{2}\tau_{2}-\xi_{2}\varepsilon_{2}}{\delta_{2}\sigma_{2}-\xi_{2}\vartheta_{2}}-L_{2}\frac{\delta_{2}\varepsilon_{2}-\vartheta_{2}\tau_{2}}{\delta_{2}\sigma_{2}-\xi_{2}\vartheta_{2}}-N_{2}\frac{\gamma_{2}\sigma_{2}\tau_{2}-\xi_{2}\varepsilon_{2}\gamma_{2}+\phi_{2}\delta_{2}\varepsilon_{2}-\phi_{2}\vartheta_{2}\tau_{2}+\delta_{2}\sigma_{2}\beta_{2}-\xi_{2}\vartheta_{2}\beta_{2}}{-2\lambda_{2}(\delta_{2}\sigma_{2}-\xi_{2}\vartheta_{2})}$$ (16)

$$s_{2}^{*}=O_{2}-V_{2}\frac{\sigma_{2}\tau_{2}-\xi_{2}\varepsilon_{2}}{\delta_{2}\sigma_{2}-\xi_{2}\vartheta_{2}}-U_{2}\frac{\delta_{2}\varepsilon_{2}-\vartheta_{2}\tau_{2}}{\delta_{2}\sigma_{2}-\xi_{2}\vartheta_{2}}-Y_{2}\frac{\gamma_{2}\sigma_{2}\tau_{2}-\xi_{2}\varepsilon_{2}\gamma_{2}+\phi_{2}\delta_{2}\varepsilon_{2}-\phi_{2}\vartheta_{2}\tau_{2}+\delta_{2}\sigma_{2}\beta_{2}-\xi_{2}\vartheta_{2}\beta_{2}}{-2\lambda_{2}(\delta_{2}\sigma_{2}-\xi_{2}\vartheta_{2})}$$ (17)

### 3.2. Second case: With simple price discount contract

Here, we use the simple price discount contracts for the proposed supply chain structure, where the wholesale price of the first manufacturer is a discounted rate of the retail price i.e. *w*_1_ = *ρp*_1_. The notations in Table 2 also are used for this case.

In this case, the demand and the profit functions are similar to before case and the retailer is leader. In the following, we investigate this case as a Retailer Stackelberg game.

#### 3.2.1. Retailer Stackelberg

In this case, we also use the conditions similar to the first case. In addition, the wholesale price of the first manufacturer is a discounted rate of the retail price.

Manufacturers Reaction Functions: The second manufacturer must choose the wholesale price $w_{2}^{*}$ and service level $s_{2}^{*}$ to maximize his own equilibrium profit and *w*_1_ = *ρp*_1_. Then, the reaction functions of the second manufacturer are:

$$w_{2}^{*}\in \arg\;\max_{w_{2}}\pi_{M_{2}}(w_{1}^{*}=\rho p_{1}^{*},w_{2},s_{2}^{*}|p_{1},p_{2},s_{1})$$ (18)

$$s_{2}^{*}\in \arg\;\max_{s_{2}}\pi_{M_{2}}(w_{1}^{*}=\rho p_{1}^{*},w_{2}^{*},s_{2}|p_{1},p_{2},s_{1})$$ (19)

where $\pi_{M_{2}}(w_{1}=\rho p_{1},w_{2},s_{2}|p_{1},p_{2},s_{1})$ given in Eq (3) denotes the profit to the second manufacturer when he sets the wholesale price *w*_2_ and the service level *s*_2_, given earlier decisions on retail prices and service level *p*_1_, *p*_2_ *and s*_1_ by the retailer. The Proposition 3 gives the results.

Proposition 3. In the Retailer Stackelberg game, for a given wholesale price *w*_1_ = *ρp*_1_ and the retail prices *p*_1_, *p*_2_ and *s*_1_, the reaction functions of the second manufacturer are derived as follows:

$$w_{2}^{*}=J_{2}-M_{2}p_{1}-L_{2}p_{2}-N_{2}s_{1}$$ (20)

$$s_{2}^{*}=O_{2}-V_{2}p_{1}-U_{2}p_{2}-Y_{2}s_{1,}$$ (21)

where *J*_2_, *M*_2_, *L*_2_, *N*_2_, *O*_2_, *V*_2_, *U*_2_ *and Y*_2_ are defined in S1 Appendix. The proof of Proposition 3 is given in S1 Appendix.

Retailer Decision: Similar to the first case, by using the reaction functions of manufacturers, we can derive the retailer's optimal retail prices and the services. In this game, the retailer must choose retail prices *p*_1_, *p*_2_ and service level *s*_1_ to maximize his equilibrium profit. That is,

$$p_{i}^{*}\in \arg\;\max_{p_{i}}\pi_{R}(p_{i},p_{j}^{*},s_{1}^{*})$$ (22)

$$s_{1}^{*}\in \arg\;\max_{s_{1}}\pi_{R}\;(p_{1}^{*},p_{j}^{*},s_{1})$$ (23)

The Proposition 4 gives the results.

Proposition 4. In the Retailer Stackelberg game, the retailer’s optimal retail prices and optimal retail service level, denoted as $p_{1}^{*},\;p_{2}^{*}$ and $s_{1}^{*}$ are given as follows:

$$p_{1}^{*}=\frac{\sigma_{3}\tau_{3}-\xi_{3}\varepsilon_{3}}{\delta_{3}\sigma_{3}-\xi_{3}\vartheta_{3}}$$ (24)

$$p_{2}^{*}=\frac{\delta_{3}\varepsilon_{3}-\vartheta_{3}\tau_{3}}{\delta_{3}\sigma_{3}-\xi_{3}\vartheta_{3}}$$ (25)

$$s_{1}^{*}=\frac{\gamma_{3}\sigma_{3}\tau_{3}-\xi_{3}\varepsilon_{3}\gamma_{3}+\phi_{3}\delta_{3}\varepsilon_{3}-\phi_{3}\vartheta_{3}\tau_{3}+\delta_{3}\sigma_{3}\beta_{3}-\xi_{3}\vartheta_{3}\beta_{3}}{-2\lambda_{3}(\delta_{3}\sigma_{3}-\xi_{3}\vartheta_{3})}$$ (26)

where *σ*_3_, *τ*_3_, *ξ*_3_, *ε*_3_, *δ*_3_ *ϑ*_3_, *γ*_3_, *ϕ*_3_, *β*_3_ and *λ*_3_ are defined in S1 Appendix.

By substituting Eq (24), Eq (25) and Eq (26) into Eq (20), Eq (21) and $w_{1}^{*}=\rho p_{1}^{*}$, the manufactures’ optimal wholesale prices and the second manufacturer optimal service level are obtained and can be shown as follows:

$$w_{1}^{*}=\rho \frac{\sigma_{3}\tau_{3}-\xi_{3}\varepsilon_{3}}{\delta_{3}\sigma_{3}-\xi_{3}\vartheta_{3}}$$ (27)

$$w_{2}^{*}=J_{2}-M_{2}\frac{\sigma_{3}\tau_{3}-\xi_{3}\varepsilon_{3}}{\delta_{3}\sigma_{3}-\xi_{3}\vartheta_{3}}-L_{2}\frac{\delta_{3}\varepsilon_{3}-\vartheta_{3}\tau_{3}}{\delta_{3}\sigma_{3}-\xi_{3}\vartheta_{3}}-N_{2}\frac{\gamma_{3}\sigma_{3}\tau_{3}-\xi_{3}\varepsilon_{3}\gamma_{3}+\phi_{3}\delta_{3}\varepsilon_{3}-\phi_{3}\vartheta_{3}\tau_{3}+\delta_{3}\sigma_{3}\beta_{3}-\xi_{3}\vartheta_{3}\beta_{3}}{-2\lambda_{3}(\delta_{3}\sigma_{3}-\xi_{3}\vartheta_{3})}$$ (28)

$$s_{2}^{*}=O_{2}-V_{2}\frac{\sigma_{3}\tau_{3}-\xi_{3}\varepsilon_{3}}{\delta_{3}\sigma_{3}-\xi_{3}\vartheta_{3}}-U_{2}\frac{\delta_{3}\varepsilon_{3}-\vartheta_{3}\tau_{3}}{\delta_{3}\sigma_{3}-\xi_{3}\vartheta_{3}}-Y_{2}\frac{\gamma_{3}\sigma_{3}\tau_{3}-\xi_{3}\varepsilon_{3}\gamma_{3}+\phi_{3}\delta_{3}\varepsilon_{3}-\phi_{3}\vartheta_{3}\tau_{3}+\delta_{3}\sigma_{3}\beta_{3}-\xi_{3}\vartheta_{3}\beta_{3}}{-2\lambda_{3}(\delta_{3}\sigma_{3}-\xi_{3}\vartheta_{3})}$$ (29)

## 4. Numerical examples

In this section, because of the complexity of the formulas, a numerical example is used to analyze scenarios in two with and without discount contract cases, as accomplished in [32]. Through this example, we examine the effect of discount rate changes on the decision variables of supply chain members and compare the results with the numerical example results without discount case. In this example, the values considered for the parameters are similar to those of the numerical examples in papers by [19–24].

### 4.1. Without discount case

In this scenario, we solve a numerical example with the following parameters: *a*_*i*_ = 40, *b*_*p*_ = 0.3, *b*_*s*_ = 0.3, *θ*_*p*_ = 0.3, *θ*_*s*_ = 0.3, *η*_*i*_ = 2, *c*_*i*_ = 2 and *i* = 1,2 (All data are available in supplementary file as (S1 File). The notations in Table 2 also are used for this case. In addition, the following notations are adopted:

***ρ*** Discount rate in second case

***π***_***M*11**_ Profit of first manufacturer in first case

***π***_***M*12**_ Profit of first manufacturer in second case

***π***_***M*21**_ Profit of second manufacturer in first case

***π***_***M*22**_ Profit of second manufacturer in second case

***π***_***R*11**_ Profit of retailer from first product in first case

***π***_***R*12**_ Profit of retailer from first product in second case

**Δ*π***_***M*1**_ Profit difference for first manufacturer between the two cases (***π***_***M*12**_ − ***π***_***M*11**_)

**Δ*π***_***M*2**_ Profit difference for second manufacturer between the two cases (***π***_***M*22**_ − ***π***_***M*21**_)

**Δ*S***_**1**_ Service level difference for retailer between the two cases

**Δ*S***_**2**_ Service level difference for second manufacturer between the two cases

**Δ*π***_***R***_ Profit difference for retailer between the two cases

**Δ*π***_***R*1**_ Difference of profit from first product for retailer between the two cases (***π***_***R*12**_ – ***π***_***R*11**_)

Table 3 shows the results of the numerical example in without discount case.

**Table 3:** Results of numerical example.

*p*_1_	93.50048672
*p*_2_	92.59643999
*w*_1_	24.46419135
*w*_2_	26.92977333
*s*_1_	6.739257405
*s*_2_	7.478931998
*Q*_1_	13.47851481
*Q*_2_	14.957864
*π*_*M*1_	302.7839358
*π*_*M*2_	316.9617351
*π*_*R*_	1867.322208

### 4.2. With discount case

In this case, we solve a numerical example with the base values of key parameters similar to the first case. (All data are available in supplementary file as (S1 File)

We analysis the effect of the changes of the discount rate ***ρ*** on the profit and the demand of supply chain's members. It is assumed that the discount rate changes and the other parameters are constant.

It should be noted, when the discount rate increases, the amount of the first manufacturer discount to the retailer decreases, because *w*_1_ = *ρp*_1_.

**Fig 3:**
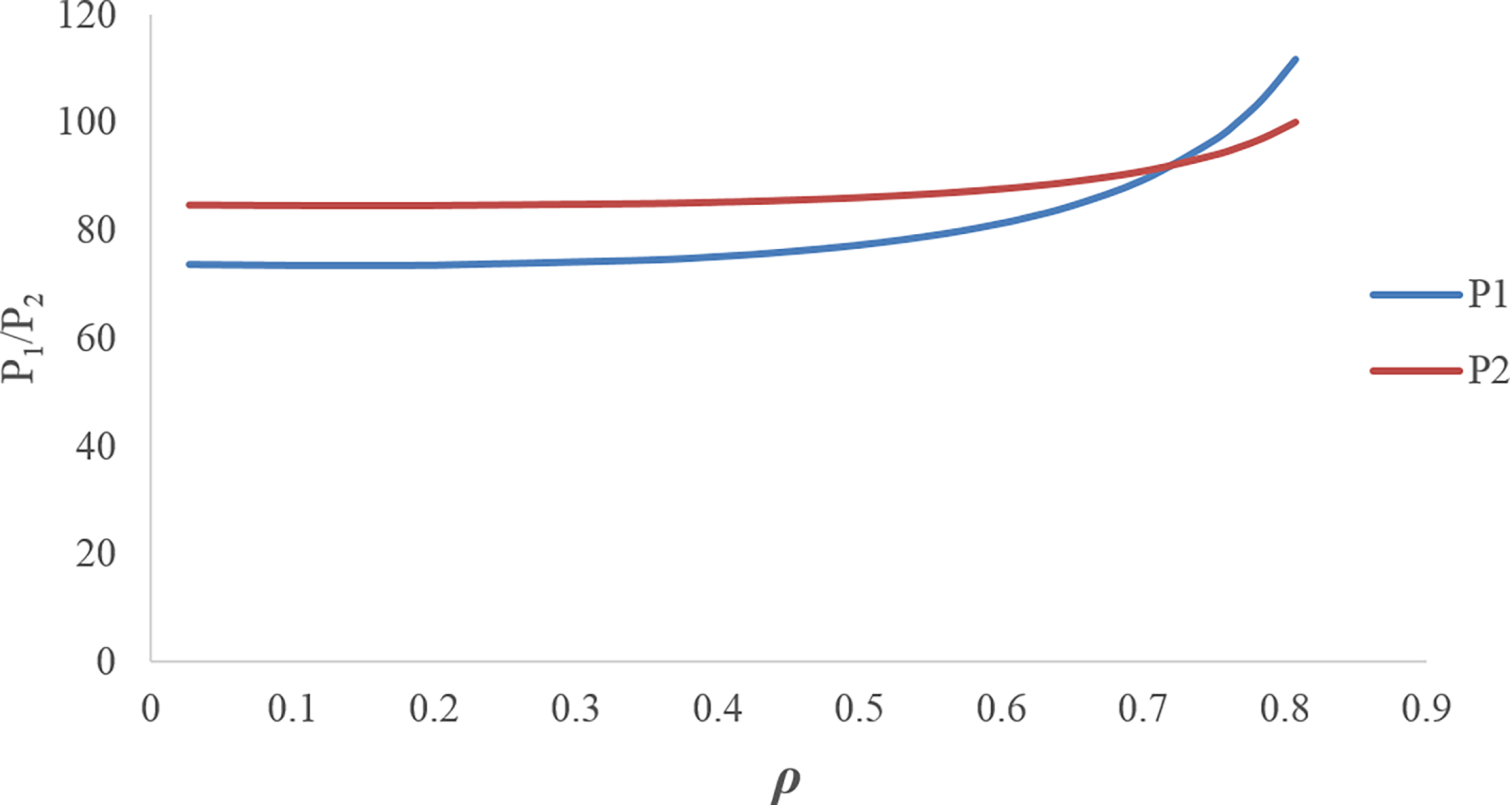
Effect of *ρ* on retail prices.

**Fig 4:**
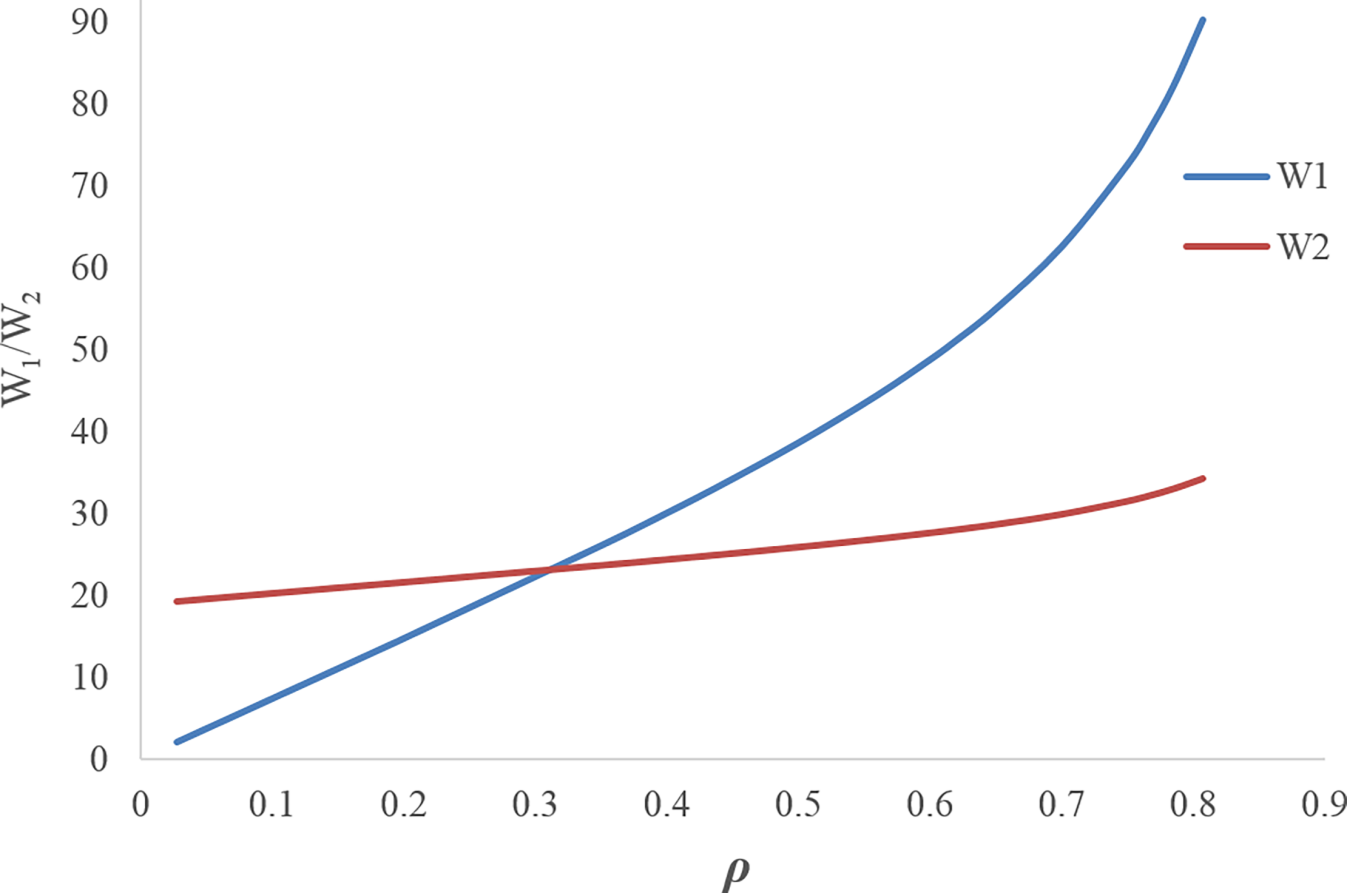
Effect of *ρ* on wholesale prices.

**Fig 5:**
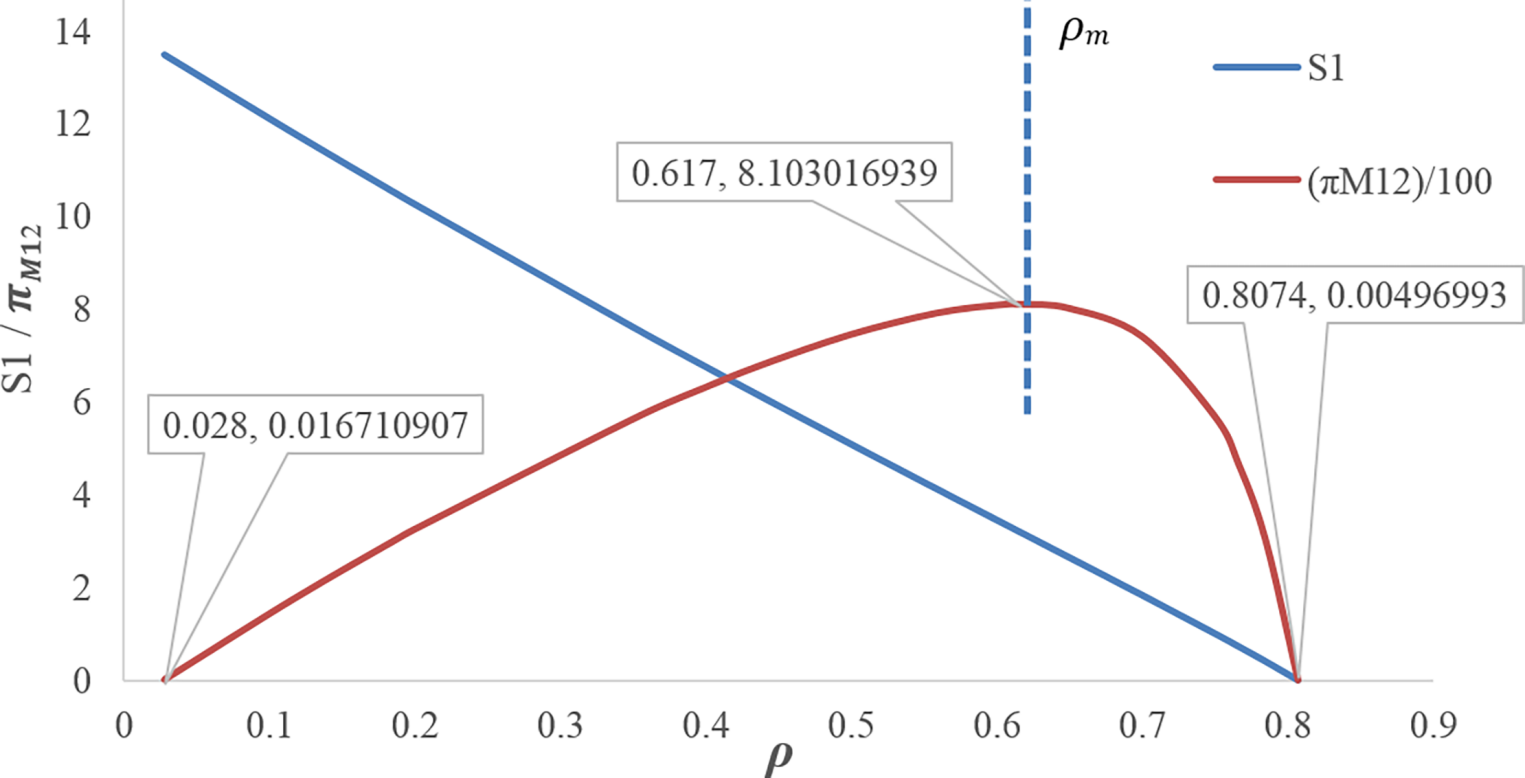
Effect of *ρ* on first service and profit of first manufacturer.

**Fig 6:**
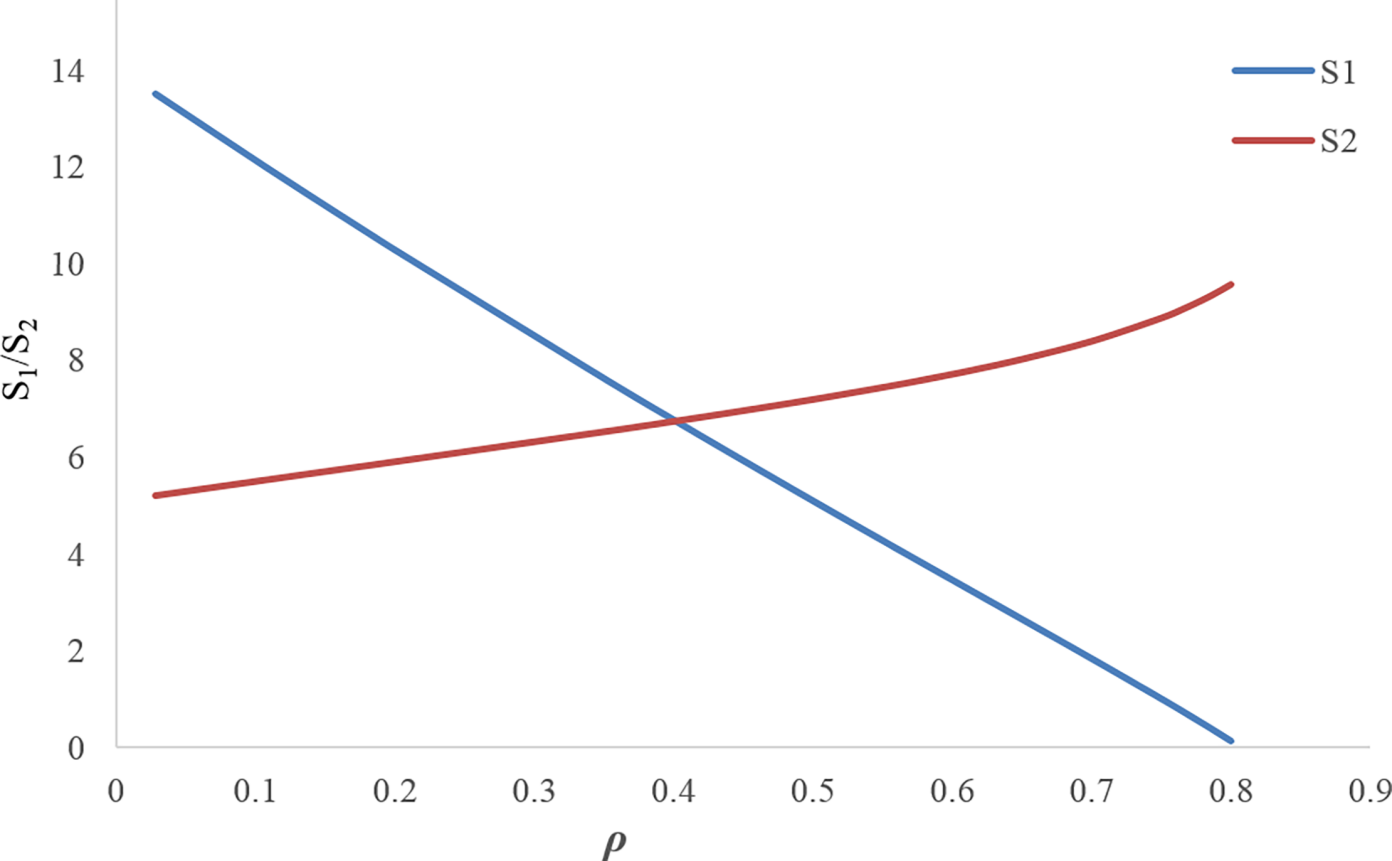
Effect of *ρ* on the service levels.

**Fig 7:**
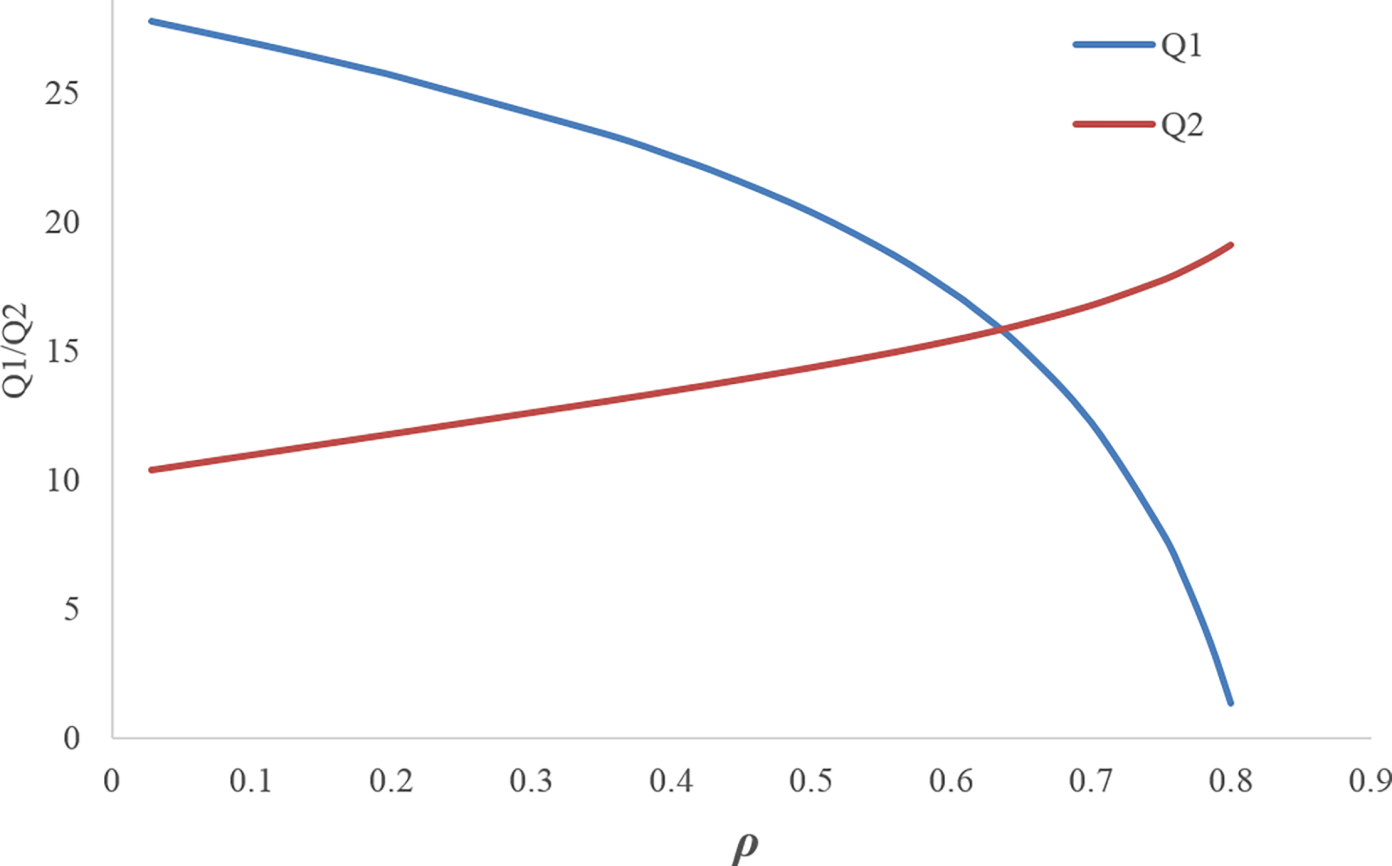
Effect of ρ on the order quantities.

**Fig 8:**
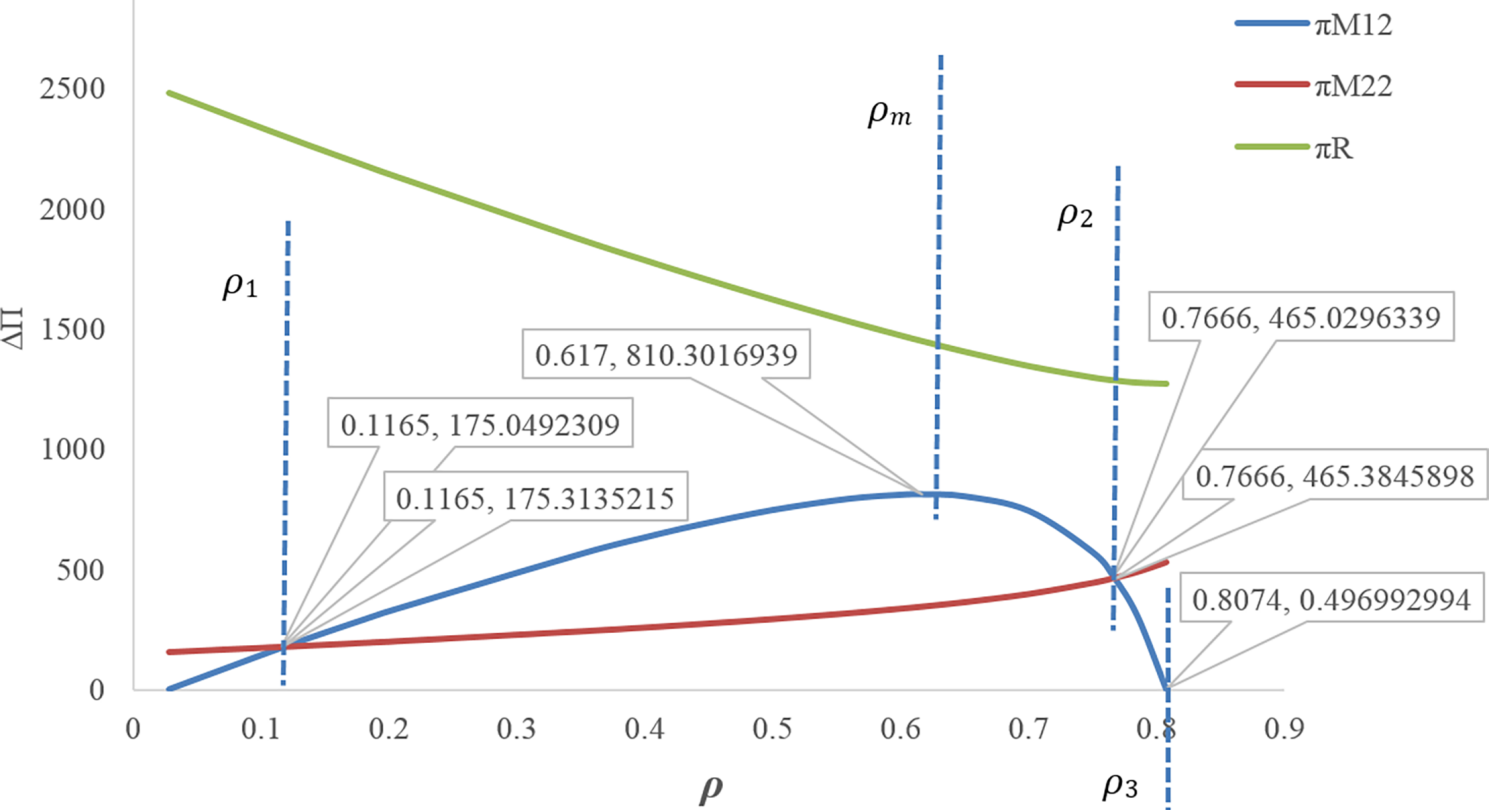
Effect of *ρ* on profits.

When the discount rate is less than or equal to 0.8 i.e. ≤0.8 and the optimal conditions are satisfied and the decision variables are positive, the results of this numerical example are obtained as follow:

1. Figs 3 and 4 illustrate that by increasing the discount rate, the retail prices and wholesale prices follow an increasing trend.
2. Fig 5 shows when the discount rate decreases (i.e. increasing the first manufacturer discount to the retailer), the amount of the first service level *s*_1_ increases, but this increasing service does not necessarily lead to an increase in the profit of the first manufacturer, because the first manufacturer's profit depends on the costs imposed on the first manufacturer due to the discount and the conditions of the other members as well. Hence, by increasing the discount rate, the first manufacturer's profit trend increases up to *ρ*_*m*_ ≅ 0.617 and then it decreases. The amount of first manufacturer's profit at the maximum point is ***π***_***M*12**_ ≅ 810.3016939. In this figure, due to the better and simultaneous presentation of the first manufacturer's profit and the first service level, ***π***_***M*12**_ is displayed on the scale of $\left(\frac{1}{100}\right)$.
3. Fig 6 shows that by increasing the discount rate, the first service level *s*_1_ follows a decreasing trend and the second service level *s*_2_ has an increasing trend.
4. When the discount rate increases, the order quantity of the first product has a decreasing trend and the order quantity of second product has an increasing trend (Fig 7).
5. By increasing the discount rate, the second manufacturer’s profit has an increasing trend and the retailer’s profit shows a decreasing trend, and the first manufacturer's profit has an increasing trend up to *ρ*_*m*_ ≅ 0.617 and then it shows a decreasing trend (Fig 8).
6. In Fig 8, if 0 ≤ *ρ* ≤ *ρ*_1_ ≅ 0.1165 or 0.7666 ≅ *ρ*_2_ ≤ *ρ* ≤ *ρ*_3_ ≅ 0.8074, the second manufacturer’s profit will be more than the first manufacturer’s profit and if 0.1165 ≅ *ρ*_1_ ≤ *ρ* ≤ *ρ*_2_ ≅ 0.7666 it’s vice versa. If *ρ* = *ρ*_1_ ≅ 0.1165 then ***π***_***M*12**_ ≅ 175.0492309 and ***π***_***M*22**_ ≅ 175.3135215. If *ρ* = *ρ*_2_ ≅ 0.7666 then ***π***_***M*12**_ ≅ 465.3845898 and ***π***_***M*22**_ ≅ 465.0296339. If *ρ* = *ρ*_3_ ≅ 0.8074 then ***π***_***M*12**_ ≅ 0.4969929.

### 4.3. Comparison of results between first case and second case

**Fig 9:**
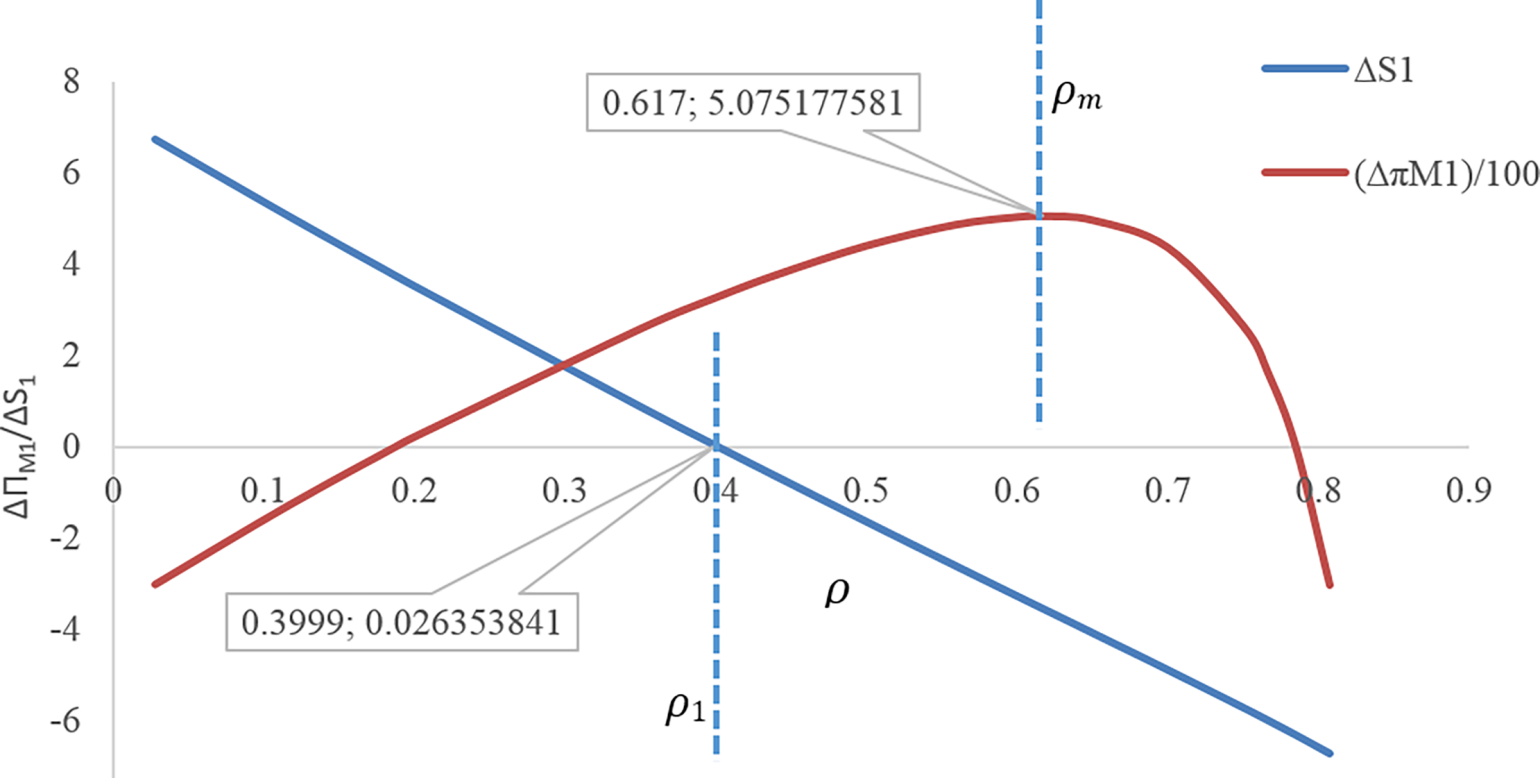
Effect of *ρ* on profits and demands.

**Fig 10:**
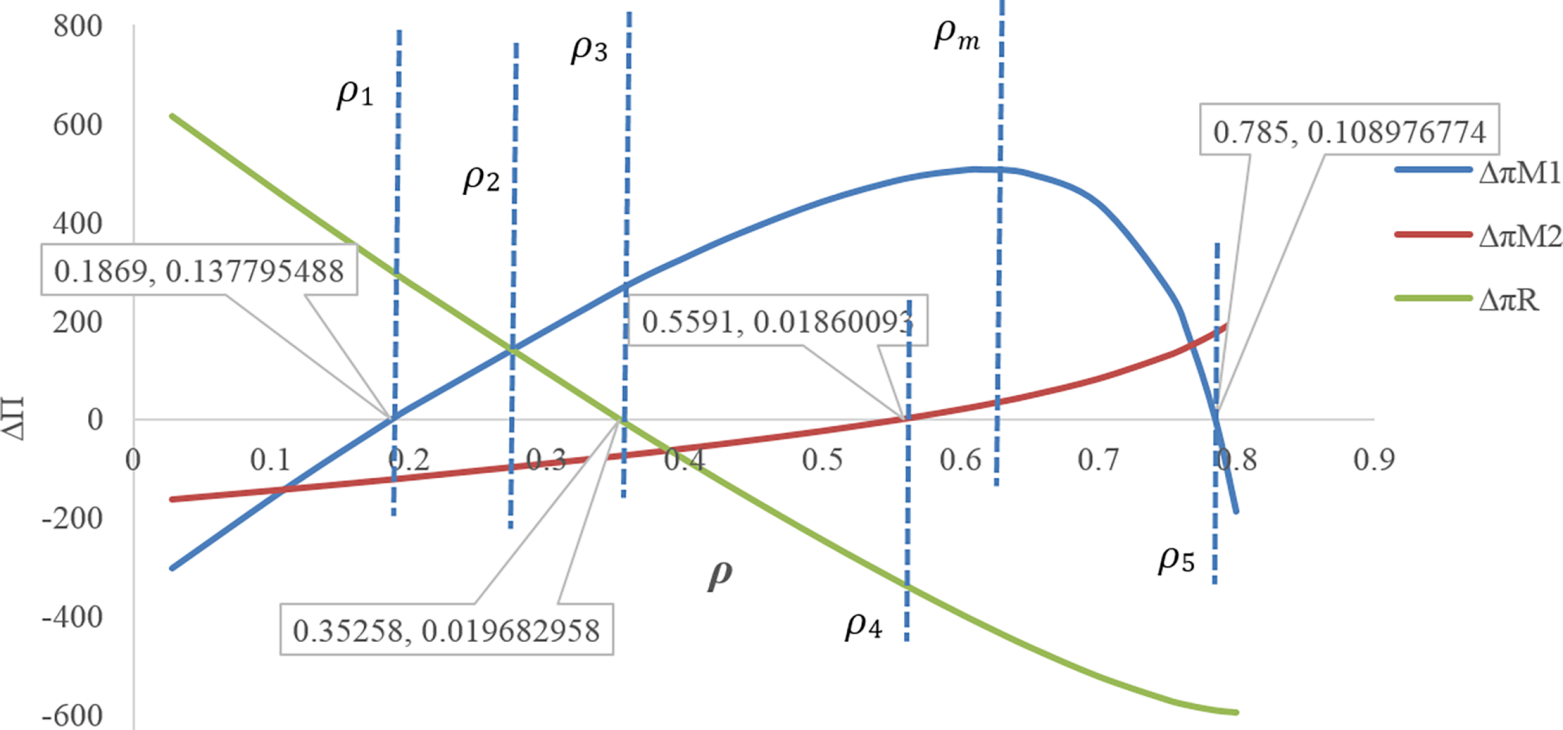
Effect of *ρ* on the profit difference for the members between two cases.

**Fig 11:**
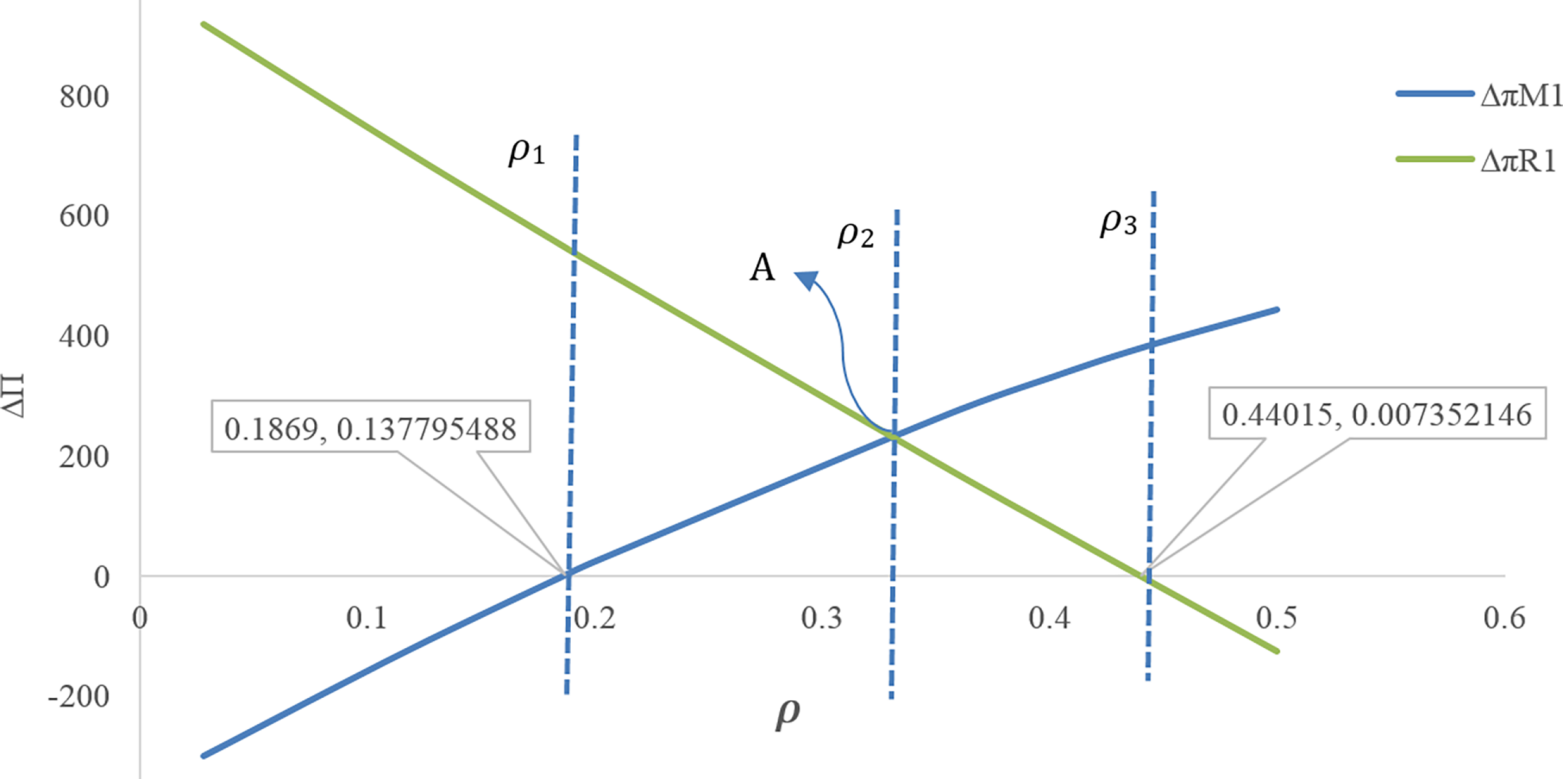
Effect of *ρ* on profit of retailer from first product and the first manufacturer’s profit.

**Fig 12:**
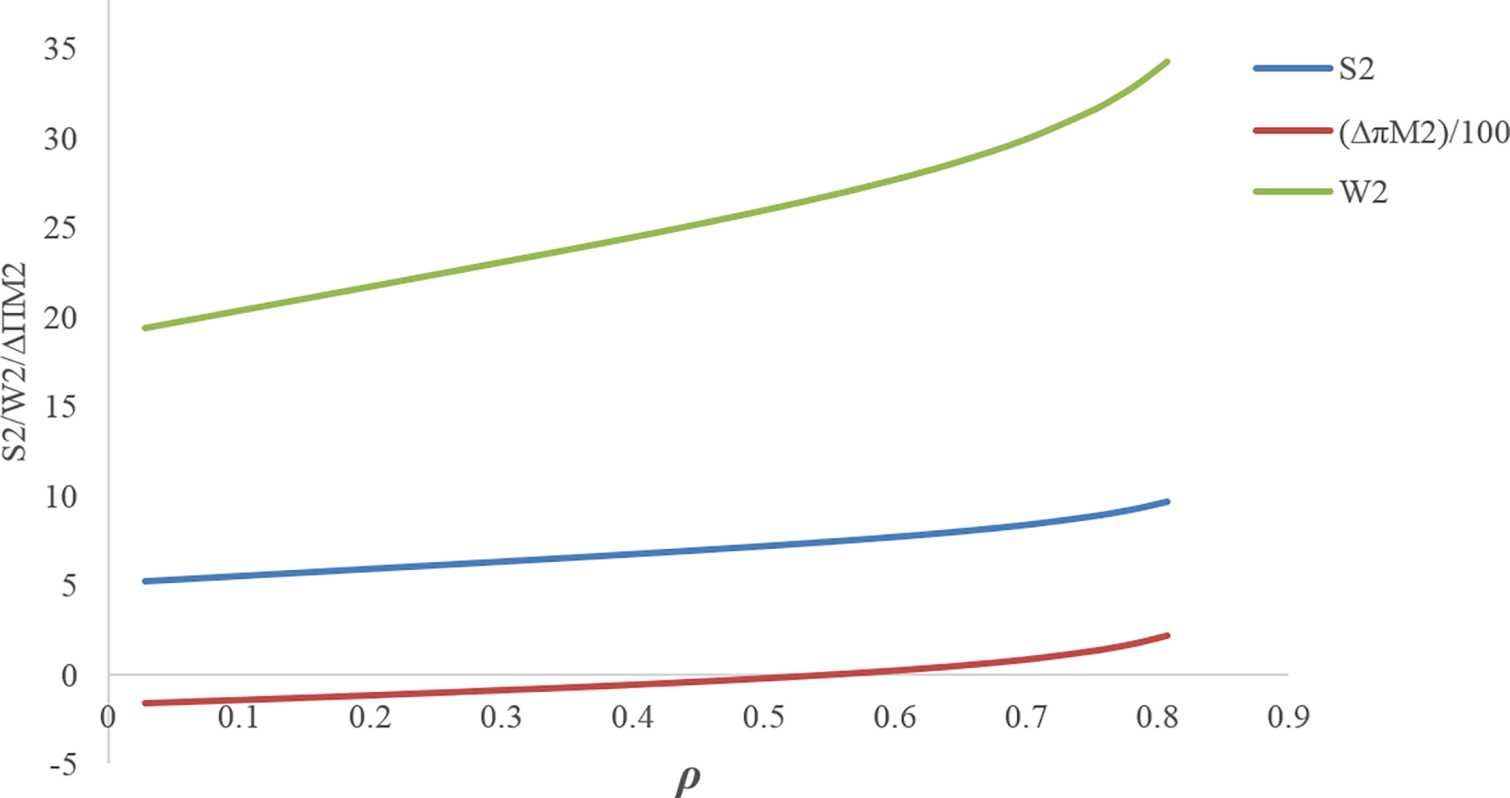
Effect of *ρ* on profit and wholesale price and service of second manufacturer.

**Fig 13:**
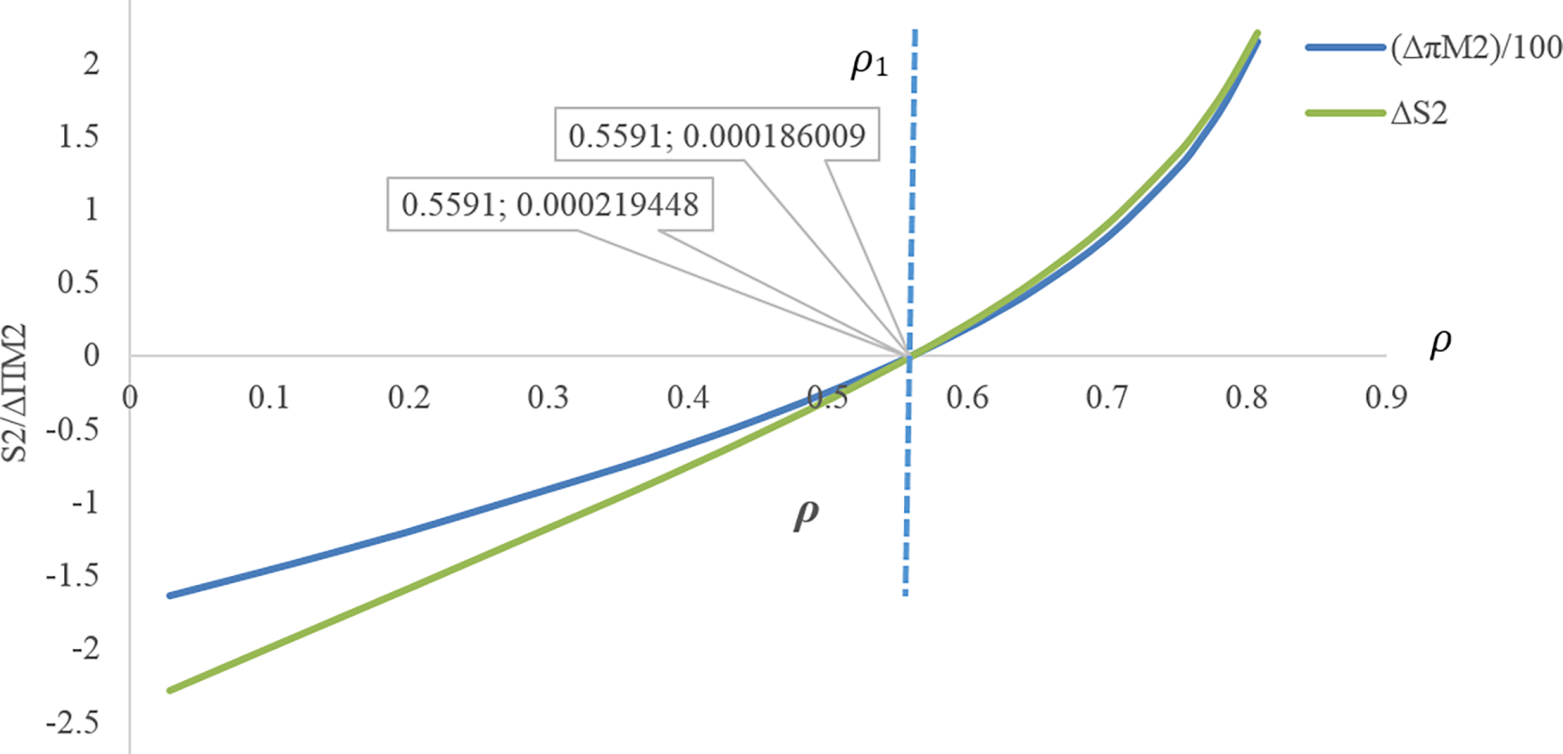
Effect of *ρ* on the service and profit of second manufacturer.

One of the objectives of this study is to examine the effects of the price discount contract on the decision variables of supply chain members. The results of comparing the numerical examples in two cases show these effects as follows (All data are available in supplementary file as (S1 File):

1. Fig 9 illustrates that at the discount rate point *ρ*_*m*_ ≅ 0.617, the profit of the first manufacturer is maximum for the discount case compared with the case without discount. In this figure, **Δ*π***_**M1**_ is displayed on the scale of $\left(\frac{1}{100}\right)$.
2. If 0 ≤ *ρ* ≤ *ρ*_1_ ≅ 0.3999, the amount of the first service level *s*_1_ will be more in the case with discount contract than when there is no discount contract, but throughout this range, the first manufacturer’s profit is not necessarily more than the case without discount contract(Fig 9).
3. If 0.1869 ≅ *ρ*_1_ ≤ *ρ* ≤ *ρ*_5_ ≅ 0.785, the first manufacturer’s profit will be more for the case with discount than the case without discount. If *ρ* = *ρ*_1_ ≅ 0.1869 then **Δ*π***_***M*1**_ ≅ 0.137795 and if *ρ* = *ρ*_5_ ≅ 0.785 then **Δ*π***_***M*1**_ ≅ 0.108976 (Fig 10).
4. If 0.5591 ≅ *ρ*_4_ ≤ *ρ*, the second manufacturer’s profit will be more in the case with discount than that in the case without discount. If *ρ* = *ρ*_4_ ≅ 0.5591 then **Δ*π***_***M*2**_ ≅ 0.0186 (Fig 10).
5. If 0.35258 ≅ *ρ*_3_ ≤ *ρ*, the retailer’s profit will be less in the case with discount than that in the without discount contract, and if 0 ≤ *ρ* ≤ *ρ*_3_ ≅ 0.35258 the opposite will happen. If *ρ* = *ρ*_3_ ≅ 0.35258 then **Δ*π***_***R***_ = 0.0196829 (Fig 10).
6. If 0.1869 ≅ *ρ*_1_ ≤ *ρ* ≤ *ρ*_3_ ≅ 0.35258, both profits of the first manufacturer and retailer will be more for the case with discount than the case with no discount contract (Fig 10).
7. If *ρ* > *ρ*_2_, the profit difference for the first manufacturer between the case with discount and the without discount is more than the profit difference for the retailer between the case with discount and the without discount, and If *ρ* < *ρ*_2_ it’s vice versa. (Fig 10)
8. There is no discount rate at which the profit of all members in the case with discount is simultaneously higher than that in the case without discount (Fig 10).
9. If 0.1869 ≅ *ρ*_1_ ≤ *ρ* ≤ *ρ*_3_ ≅ 0.44015, both of the first manufacturer’s profit and the profit of the retailer from the first product will be more in the case with discount than when there is no discount contract. If *ρ* ≥ *ρ*_1_ ≅ 0.1869 then **Δ*π***_***M*1**_ ≥ 0.13779, and If *ρ* ≤ *ρ*_3_ ≅ 0.44015 then **Δ*π***_***R*1**_ ≥ 0.007352, but if 0.1869 ≅ *ρ*_1_ ≤ *ρ* ≤ *ρ*_3_ ≅ 0.44015 then (**Δ*π***_***M*1**_ and **Δ*π***_***R*1**_) ≥ 0 (Fig 11).
10. Fig 11 shows that point (A) (i.e. when *ρ* = *ρ*_2_) can be a point to coordinate of price discount contract and retail service decisions between the first manufacturer and the retailer, while the profit difference for both of them i.e. **Δ*π***_***M*1**_ and **Δ*π***_***R*1**_ will be maximum at the same time. If *ρ* > *ρ*_2_ then **Δ*π***_***M*1**_ < **Δ*π***_***R*1**_, and If *ρ* > *ρ*_2_ then **Δ*π***_***M*1**_ > **Δ*π***_***R*1**_.
11. By decreasing the discount rate or increasing the amount of discounts provided by the first manufacturer for the retailer, the service level provided by the second manufacturer for the customer as well as his wholesale price and profit decreases. In fact, it is concluded that when the first manufacturer applies more discount, according to the situation and the intensity of competition, the second manufacturer will have to choose a lower wholesale price. It also reduces its service costs. Moreover, it can also be concluded that as the first manufacturer applies less discounts for the retailer, the second manufacturer will provide more services, which leads to an increase in the his profit (Fig 12). In this figure, **Δ*π***_***M*2**_ is displayed on the scale of $\left(\frac{1}{100}\right)$.
12. If 0.5591 ≅ *ρ*_1_ ≤ *ρ* ≤ 0.8, the amount of the second service level S_2_ and the second manufacturer's profit will be more in the case with discount contract than when there is no discount contract. If *ρ* = *ρ*_1_ ≅ 0.5591 then **Δ*π***_***M*2**_ ≅ 0.000186 and **Δ*S***_**2**_ = 0.000219 (Fig 13).

The results of the numerical examples are consistent with the results of papers such as [18–19–32]. More insights are gained based on the results of the above numerical examples. If the first manufacturer and the retailer make coordinated decisions, they can always gain more profit in the second case than the first case.

## 5. Conclusion

We have examined the competition in a supply chain consisting two manufacturers and one retailer under three factors including price, service and simple price discount contract. We have assumed the second manufacturer provides service directly to his customers, and the retailer provides service to the first product customers, while he buys the first product with price discount. By using game theoretic approach, we have derived equilibrium solutions of Retailer Stackelberg games. We have compared and analyzed the results of numerical examples in two case: **a)** the manufacturers sell their products to retailer without price discount contract. **b)** The first manufacturer sells his products to retailer with the simple price discount contract. We have shown that services and discounts had high impacts on the profit of supply chain members, and if a manufacturer chooses appropriate discount rates and sells the product with simple price discount contract, there will be higher profit compared with the case without discount contract. This situation can be considered as an effective tool for the coordination of the first manufacturer and retailer to offer discount and provide the service.

The obtained results of sensitivity analysis of numerical examples show that service and price discount contract can improve the performance of supply chain. Several scenarios are possible for future research. First, competition in a supply chain with structure differs of our supply chain structure. Second, we have examined he competition in a supply chain under service and other type of discounts.

The choice of discount rate range by the manufacturer is very important because offering any amount of discount rate does not always lead to an increase in the manufacturer's profit. At some discount rate intervals, the manufacturer's revenue will be lowered, which should not be chosen by the manufacturer. In addition, there is a unique discounted rate, where the manufacturer's profit is maximal, and a managerial decision can make that point. In real life, the proposed model can be used more effectively in the production and sales of electronic and computer products. In future research, the supply chain and power structure can be considered different from this study.

## Supporting information

S1 Appendix The details of the proof of the propositions. (DOCX)

S1 File The details of data and calculations of numerical examples. (RAR)
