# Supplementary material for: Retailer Stackelberg game in a supply chain with pricing and service decisions and simple price discount contract
Source: PLoS One. 2018 Apr 12;13(4):e0195109. doi: 10.1371/journal.pone.0195109 (PMC5896922; doi:10.1371/journal.pone.0195109)
Supplement: S1 Appendix — (DOCX) [file pone.0195109.s001.docx]

**S1 Appendix**

**Proof of Proposition 1**. By solving the ﬁrst conditions $\frac{\partial\pi_{Mi}}{\partial w_{i}}=0$ and $\frac{\partial\pi_{M2}}{\partial s_{2}}=0$ , the manufacturers reaction functions are obtained as:

$\left( b_{p}+\theta_{p} \right)w_{1}+\theta_{s}s_{2}=a_{1}-\left( b_{p}+\theta_{p} \right)p_{1}+\theta_{p}p_{2}\boldsymbol{+}\left( b_{s}\boldsymbol{+}\theta_{s} \right)s_{1}+\left( b_{p}+\theta_{p} \right)c_{1}$ (30)

$\left( b_{p}+\theta_{p} \right)w_{2}-\left( b_{s}\boldsymbol{+}\theta_{s} \right)s_{2}=a_{2}-\left( b_{p}+\theta_{p} \right)p_{2}+\theta_{p}p_{1}-\theta_{s}s_{1}+\left( b_{p}+\theta_{p} \right)c_{2}$ (31)

$\left( b_{s}\boldsymbol{+}\theta_{s} \right)w_{2}-\eta_{2}s_{2}=\left( b_{s}\boldsymbol{+}\theta_{s} \right)c_{2}$ (32)

By assuming $p_{1}=w_{1}+m_{1}$**,** $p_{2}=w_{2}+m_{2}$ **,**  $A=-2\left( b_{p}+\theta_{p} \right)$ $B=\left( b_{s}+\theta_{s} \right)$ , $D_{2}=a_{1}+0.5Ap_{1}+\theta_{p}p_{2}\boldsymbol{+}Bs_{1}-0.5Ac_{1}$ , $E_{2}=a_{2}+0.5Ap_{2}+\theta_{p}p_{1}-\theta_{s}s_{1}-0.5Ac_{2}$ ,equations (30), (31), and (32) reduces to equations (33), (34), and (35) respectively:

$-0.5Aw_{1}+\theta_{s}s_{2}=D_{2}$ (33), $-0.5Aw_{2}-Bs_{2}=E_{2}$ (34), $Bw_{2}-\eta_{2}s_{2}=Bc_{2}$ (35)

By solving (33), (34) and (35), simultaneously, we get:

$w_{1}=\frac{-0.5A\eta_{2}D_{2}-D_{2}B^{2}-B\theta_{s}E_{2}-0.5A{B\theta_{s}c}_{2}}{(0.5A)(0.5A\eta_{2}+B^{2})}$ (36), $w_{2}=\frac{B^{2}c_{2}-\eta_{2}E_{2}}{0.5A\eta_{2}+B^{2}}$ (37), $s_{2}=\frac{-BE_{2}-0.5ABc_{2}}{0.5A\eta_{2}+B^{2}}$ (38)

By assuming $F_{2}=\left( 0.5A\eta_{2}+B^{2} \right)$ , $I_{2}=\left( \frac{1}{0.5AF_{2}} \right)\left( -a_{1}F_{2}+0.5{AF}_{2}c_{1}-a_{2}B\theta_{s} \right)$, $G_{2}=\left( \frac{1}{0.5AF_{2}} \right)\left( 0.5{AF}_{2}+B\theta_{s}\theta_{p} \right)$, $H_{2}=\left( \frac{1}{0.5AF_{2}} \right)\left( F_{2}\theta_{p}+0.5AB\theta_{s} \right)$ , $K_{2}=\left( \frac{1}{0.5AF_{2}} \right)\left( F_{2}B-B\theta_{s}^{2} \right)$,

$J_{2}=\frac{B^{2}c_{2}-{a_{2}\eta}_{2}+0.5Ac_{2}\eta_{2}}{F_{2}}$ , $L_{2}=\frac{0.5A\eta_{2}}{F_{2}}$ , $M_{2}=\frac{\eta_{2}\theta_{p}}{F_{2}}$ , $N_{2}=-\frac{\eta_{2}\theta_{s}}{F_{2}}$, $O_{2}=-\frac{a_{2}B}{F_{2}}$ , $U_{2}=\frac{0.5AB}{F_{2}}$ , $V_{2}=\frac{B\theta_{p}}{F_{2}}$ , $Y_{2}=-\frac{B\theta_{s}}{F_{2}}$, equations (36), (37), and (38) reduces to equations (39), (40), and (41) respectively:

$w_{1}^{*}=I_{2}-G_{2}p_{1}-H_{2}p_{2}-K_{2}s_{1}$ (39) $w_{2}^{*}=J_{2}-M_{2}p_{1}-L_{2}p_{2}-N_{2}s_{1}$ (40)

$s_{2}^{*}=O_{2}-V_{2}p_{1}-U_{2}p_{2}-Y_{2}s_{1}$ (41)

Taking the second-order partial derivatives of $\pi_{M2}$ with respect to $w_{2}$ and $s_{2}$, we have optimal second condition and the Hessian Matrix, respectively,

$\frac{\partial^{2}\pi_{M2}}{\partial{w_{2}}^{2}}=-\left( b_{p}+\theta_{p} \right)<0$ , $H=\left[ \begin{matrix} \frac{\partial^{2}\pi_{M2}}{\partial{w_{2}}^{2}} & \frac{\partial^{2}\pi_{M2}}{\partial w_{2}\partial s_{2}} \\ \frac{\partial^{2}\pi_{M2}}{\partial s_{2}\partial w_{2}} & \frac{\partial\pi_{M2}}{\partial s_{2}} \end{matrix} \right]=\left[ \begin{matrix} -\left( b_{p}+\theta_{p} \right) & \left( b_{s}\boldsymbol{+}\theta_{s} \right) \\ \left( b_{s}\boldsymbol{+}\theta_{s} \right) & -\eta_{2} \end{matrix} \right]$

${(H}_{11}=-\left( b_{p}+\theta_{p} \right)<0)$ , $Det\left( H \right)=\left( \left( b_{p}+\theta_{p} \right)\eta_{2}-\left( b_{s}\boldsymbol{+}\theta_{s} \right)^{2} \right)>0$

**Proof of Proposition 2.** In this stage, by substituting Eqs. (39), (40), (41) in $\pi_{R}$ and solving the ﬁrst conditions $\frac{\partial\pi_{R}}{\partial p_{i}}=0$ and $\frac{\partial\pi_{R}}{\partial s_{1}}=0$ , the retailer's reaction functions are obtained as:

$\frac{\partial\pi_{R}}{\partial p_{1}}=R_{2}+2T_{2}p_{1}+X_{2}p_{2}+\gamma_{2}s_{1}=0$ (42)*,* $\frac{\partial\pi_{R}}{\partial p_{2}}=Z_{2}+X_{2}p_{1}+2\alpha_{2}p_{2}+\phi_{2}s_{1}=0$ (43)

$\frac{\partial\pi_{R}}{\partial s_{1}}=\beta_{2}+\gamma_{2}p_{1}+2\lambda_{2}s_{1}+\phi_{2}p_{2}=0$ (44)

By assuming $R_{2}=\left[ a_{1}\left( 1+G_{2} \right)+I_{2}\left( b_{p}+\theta_{p} \right)-\left( 1+G_{2} \right)\theta_{s}O_{2}-I_{2}\theta_{s}V_{2}+a_{2}M_{2}-J_{2}\theta_{p}+M_{2}\left( b_{s}+\theta_{s} \right)O_{2}+J_{2}\left( b_{s}+\theta_{s} \right)V_{2} \right]$ , $T_{2}=\left[ -\left( 1+G_{2} \right)\left( b_{p}+\theta_{p} \right)+\left( 1+G_{2} \right)\theta_{s}V_{2}+M_{2}\theta_{p}-M_{2}\left( b_{s}+\theta_{s} \right)V_{2} \right]$ , $X_{2}=\left[ -H_{2}\left( b_{p}+\theta_{p} \right)+\left( 1+G_{2} \right)\theta_{p}+\left( 1+G_{2} \right)\theta_{s}U_{2}+H_{2}\theta_{s}V_{2}-M_{2}\left( b_{p}+\theta_{p} \right)+\left( 1+L_{2} \right)\theta_{p}-M_{2}\left( b_{s}+\theta_{s} \right)U_{2}-\left( 1+L_{2} \right)\left( b_{s}+\theta_{s} \right)V_{2} \right]$ , $\gamma_{2}=\left[ -K_{2}\left( b_{p}+\theta_{p} \right)+K_{2}\theta_{s}V_{2}+\left( 1+G_{2} \right)\theta_{s}Y_{2}+N_{2}\theta_{p}-N_{2}\left( b_{s}+\theta_{s} \right)V_{2}-M_{2}\left( b_{s}+\theta_{s} \right)Y_{2} \right]$ , $Z_{2}=\left[ a_{1}H_{2}-I_{2}\theta_{p}-H_{2}\theta_{s}O_{2}-I_{2}\theta_{s}U_{2}+a_{2}\left( 1+L_{2} \right)+J_{2}\left( b_{p}+\theta_{p} \right)+\left( 1+L_{2} \right)\left( b_{s}+\theta_{s} \right)O_{2}+J_{2}\left( b_{s}+\theta_{s} \right)U_{2} \right]$ , $\alpha_{2}=\left[ H_{2}\theta_{p}-H_{2}\theta_{s}U_{2}-\left( 1+L_{2} \right)\left( b_{p}+\theta_{p} \right)-\left( 1+L_{2} \right)\left( b_{s}+\theta_{s} \right)U_{2} \right]$, $\phi_{2}=\left[ K_{2}\theta_{p}+H_{2}\left( b_{s}+\theta_{s} \right)+K_{2}\theta_{s}U_{2}+H_{2}\theta_{s}Y_{2}-N_{2}\left( b_{p}+\theta_{p} \right)-N_{2}\left( b_{s}+\theta_{s} \right)U_{2}-\left( 1+L_{2} \right)\left( b_{s}+\theta_{s} \right)Y_{2} \right]$, $\beta_{2}=\left[ a_{1}K_{2}-I_{2}\left( b_{s}+\theta_{s} \right)-K_{2}\theta_{s}O_{2}-I_{2}\theta_{s}Y_{2}+a_{2}N_{2}+N_{2}\left( b_{s}+\theta_{s} \right)O_{2}+J_{2}\left( b_{s}+\theta_{s} \right)Y_{2} \right]$, $\lambda_{2}=\left[ K_{2}\left( b_{s}+\theta_{s} \right)+K_{2}\theta_{s}Y_{2}-\frac{\eta_{1}}{2}-N_{2}\left( b_{s}+\theta_{s} \right)Y_{2} \right]$

By solving (42), (43) and (44) simultaneously, and assuming$\delta_{2}=X_{2}\gamma_{2}-2T_{2}\phi_{2}$, $\xi_{2}=2\alpha_{2}\gamma_{2}-\phi_{2}X_{2}$ , $\tau_{2}=R_{2}\phi_{2}-Z_{2}\gamma_{2}$ , $\vartheta_{2}=\phi_{2}\gamma_{2}-2{\lambda_{2}X}_{2}$ , $\sigma_{2}=\phi_{2}^{2}-4\alpha_{2}\lambda_{2}$ *,* $\varepsilon_{2}=2\lambda_{2}Z_{2}-\phi_{2}\beta_{2}$ *,* $\delta_{2}p_{1}+\xi_{2}p_{2}=\tau_{2}$ *,* $\vartheta_{2}p_{1}+\sigma_{2}p_{2}=\varepsilon_{2}$ *,*  we get:

$p_{1}=\frac{\sigma_{2}\tau_{2}-\xi_{2}\varepsilon_{2}}{\delta_{2}\sigma_{2}-\xi_{2}\vartheta_{2}}$ *,* $p_{2}=\frac{\delta_{2}\varepsilon_{2}-\vartheta_{2}\tau_{2}}{\delta_{2}\sigma_{2}-\xi_{2}\vartheta_{2}}$

$$s_{1}=\frac{\gamma_{2}\sigma_{2}\tau_{2}-\xi_{2}\varepsilon_{2}\gamma_{2}+\phi_{2}\delta_{2}\varepsilon_{2}-\phi_{2}\vartheta_{2}\tau_{2}+\delta_{2}\sigma_{2}\beta_{2}-\xi_{2}\vartheta_{2}\beta_{2}}{-2\lambda_{2}(\delta_{2}\sigma_{2}-\xi_{2}\vartheta_{2})}$$

Taking the second-order partial derivatives of $\pi_{R}$ with respect to $p_{1}, p_{2}$ and $s_{1}$, respectively, we have the Hessian Matrix:

$$H=\left[ \begin{matrix} \frac{\partial^{2}\pi_{R}}{\partial{p_{1}}^{2}} & \frac{\partial^{2}\pi_{R}}{\partial p_{1}\partial p_{2}} & \frac{\partial^{2}\pi_{R}}{\partial p_{1}\partial s_{1}} \\ \frac{\partial^{2}\pi_{R}}{\partial p_{2}\partial p_{1}} & \frac{\partial^{2}\pi_{R}}{\partial{p_{2}}^{2}} & \frac{\partial^{2}\pi_{R}}{\partial p_{2}\partial s_{1}} \\ \frac{\partial^{2}\pi_{R}}{\partial s_{1}\partial p_{1}} & \frac{\partial^{2}\pi_{R}}{\partial s_{1}\partial p_{2}} & \frac{\partial^{2}\pi_{R}}{\partial{s_{1}}^{2}} \end{matrix} \right]=\left[ \begin{matrix} {2T}_{2} & X_{2} & \gamma_{2} \\ X_{2} & 2\alpha_{2} & \phi_{2} \\ \gamma_{2} & \phi_{2} & 2\lambda_{2} \end{matrix} \right]$$

Since, the second order optimization conditions, can be derived:

$\mathrm{if} {(H}_{11}=\frac{\partial^{2}\pi_{R}}{\partial{p_{1}}^{2}}={2T}_{2}<0), {\det\left( \left[ \begin{matrix} {2T}_{2} & X_{2} \\ X_{2} & 2\alpha_{2} \end{matrix} \right] \right)=4\alpha_{2}T_{2}}-X_{2}^{2}>0 \mathrm{and} \det\left( H \right)=[2T_{2}\left( 4\alpha_{2}\lambda_{2}-\phi_{s}^{2} \right)-X_{2}\left( 2X_{2}\lambda_{2}-\gamma_{2}\phi_{2} \right)+\gamma_{2}\left( X_{2}\phi_{2}-2\alpha_{2}\gamma_{2} \right)]<0 ,$ $\pi_{R} is strictly concave in p_{1}, p_{2}\mathrm{and}s_{1}$.

**Proof of Proposition 3**. Similar to Proof of Proposition 1, By solving the ﬁrst conditions $\frac{\partial\pi_{M2}}{\partial w_{2}}=0$ and $\frac{\partial\pi_{M2}}{\partial s_{2}}=0$ , the manufacturers reaction functions are obtained as:

${w_{1}=\rho p}_{1}$ (45) , $w_{2}=\frac{B^{2}c_{2}-\eta_{2}E_{2}}{0.5A\eta_{2}+B^{2}}$ (46) , $s_{2}=\frac{-BE_{2}-0.5ABc_{2}}{0.5A\eta_{2}+B^{2}}$ (47)

Equations (45), (46), and (47) reduces to equations (48), (49), and (50) respectively:

$w_{1}^{*}=\rho p_{1}$ (48) $w_{2}^{*}=J_{2}-M_{2}p_{1}-L_{2}p_{2}-N_{2}s_{1}$ (49)

$s_{2}^{*}=O_{2}-V_{2}p_{1}-U_{2}p_{2}-Y_{2}s_{1}$ (50)

The second-order partial derivatives of $\pi_{M2}$ with respect to $w_{2}$ and $s_{2}$ are Similar to Proof of Proposition1.

**Proof of Proposition 4.** In this stage, by substituting Eqs. (48), (49), (50) in $\pi_{R}$ and solving the ﬁrst conditions $\frac{\partial\pi_{R}}{\partial p_{i}}=0$ and $\frac{\partial\pi_{R}}{\partial s_{1}}=0$ , the retailer's reaction functions are obtained as:

$\frac{\partial\pi_{R}}{\partial p_{1}}=R_{3}+2T_{3}p_{1}+X_{3}p_{2}+\gamma_{3}s_{1}=0$ (51) *,* $\frac{\partial\pi_{R}}{\partial p_{2}}=Z_{3}+X_{3}p_{1}+2\alpha_{3}p_{2}+\phi_{3}s_{1}=0$ (52)

$\frac{\partial\pi_{R}}{\partial s_{1}}=\beta_{3}+\gamma_{3}p_{1}+2\lambda_{3}s_{1}+\phi_{3}p_{2}=0$ (53)

By assuming $R_{3}=\left[ a_{1}\left( 1-\rho\right)-\left( 1-\rho\right)\theta_{s}O_{2}+a_{2}M_{2}-J_{2}\theta_{p}+M_{2}\left( b_{s}+\theta_{s} \right)O_{2}+J_{2}\left( b_{s}+\theta_{s} \right)V_{2} \right]$, $T_{3}=\left[ -\left( 1-\rho\right)\left( b_{p}+\theta_{p} \right)+\left( 1-\rho\right)\theta_{s}V_{2}+M_{2}\theta_{p}-M_{2}\left( b_{s}+\theta_{s} \right)V_{2} \right]$ , $X_{3}=\left[ \left( 1-\rho\right)\theta_{p}+\left( 1-\rho\right)\theta_{s}U_{2}-M_{2}\left( b_{p}+\theta_{p} \right)+\left( 1+L_{2} \right)\theta_{p}-M_{2}\left( b_{s}+\theta_{s} \right)U_{2}-\left( 1+L_{2} \right)\left( b_{s}+\theta_{s} \right)V_{2} \right]$, $\gamma_{3}=\left[ \left( 1-\rho\right)\left( b_{s}+\theta_{s} \right)+\left( 1-\rho\right)\theta_{s}Y_{2}+N_{2}\theta_{p}-N_{2}\left( b_{s}+\theta_{s} \right)V_{2}-M_{2}\left( b_{s}+\theta_{s} \right)Y_{2}-M_{2}\theta_{s} \right]$, $Z_{3}=\left[ a_{2}\left( 1+L_{2} \right)+J_{2}\left( b_{p}+\theta_{p} \right)+\left( 1+L_{2} \right)\left( b_{s}+\theta_{s} \right)O_{2}+J_{2}\left( b_{s}+\theta_{s} \right)U_{2} \right]$ , $\alpha_{2}=\left[ -\left( 1+L_{2} \right)\left( b_{p}+\theta_{p} \right)-\left( 1+L_{2} \right)\left( b_{s}+\theta_{s} \right)U_{2} \right]$ , $\phi_{2}=\left[ -N_{2}\left( b_{p}+\theta_{p} \right)-N_{2}\left( b_{s}+\theta_{s} \right)U_{2}-\left( 1+L_{2} \right)\left( b_{s}+\theta_{s} \right)Y_{2}-\left( 1+L_{2} \right)\theta_{s} \right]$ , $\beta_{2}=\left[ a_{2}N_{2}+N_{2}\left( b_{s}+\theta_{s} \right)O_{2}+J_{2}\left( b_{s}+\theta_{s} \right)Y_{2}+J_{2}\theta_{s} \right]$ , $\lambda_{2}=\left[ -\frac{\eta_{1}}{2}-N_{2}\left( b_{s}+\theta_{s} \right)Y_{2}-N_{2}\theta_{s} \right]$

By solving (51), (52) and (53) simultaneously, and assuming $\delta_{3}=X_{3}\gamma_{3}-2T_{3}\phi_{3}$, $\xi_{3}=2\alpha_{3}\gamma_{3}-\phi_{3}X_{3}$ , $\tau_{3}=R_{3}\phi_{3}-Z_{3}\gamma_{3}$ , $\vartheta_{3}=\phi_{3}\gamma_{3}-2{\lambda_{3}X}_{3}$ , $\sigma_{3}=\phi_{3}^{2}-4\alpha_{3}\lambda_{3}$ *,* $\varepsilon_{3}=2\lambda_{3}Z_{3}-\phi_{3}\beta_{3}$ *,* $\delta_{3}p_{1}+\xi_{3}p_{2}=\tau_{3}$ *,* $\vartheta_{3}p_{1}+\sigma_{3}p_{2}=\varepsilon_{3}$ *,*  we get:

$p_{1}^{*}=\frac{\sigma_{3}\tau_{3}-\xi_{3}\varepsilon_{3}}{\delta_{3}\sigma_{3}-\xi_{3}\vartheta_{3}}$ (54) $p_{2}^{*}=\frac{\delta_{3}\varepsilon_{3}-\vartheta_{3}\tau_{3}}{\delta_{3}\sigma_{3}-\xi_{3}\vartheta_{3}}$ (55)

$s_{1}^{*}=\frac{\gamma_{3}\sigma_{3}\tau_{3}-\xi_{3}\varepsilon_{3}\gamma_{3}+\phi_{3}\delta_{3}\varepsilon_{3}-\phi_{3}\vartheta_{3}\tau_{3}+\delta_{3}\sigma_{3}\beta_{3}-\xi_{3}\vartheta_{3}\beta_{3}}{-2\lambda_{3}(\delta_{3}\sigma_{3}-\xi_{3}\vartheta_{3})}$ (56)

Taking the second-order partial derivatives of $\pi_{R}$ with respect to $p_{1}, p_{2}$ and $s_{1}$, respectively, we have the Hessian Matrix:

$$H=\left[ \begin{matrix} \frac{\partial^{2}\pi_{R}}{\partial{p_{1}}^{2}} & \frac{\partial^{2}\pi_{R}}{\partial p_{1}\partial p_{2}} & \frac{\partial^{2}\pi_{R}}{\partial p_{1}\partial s_{1}} \\ \frac{\partial^{2}\pi_{R}}{\partial p_{2}\partial p_{1}} & \frac{\partial^{2}\pi_{R}}{\partial{p_{2}}^{2}} & \frac{\partial^{2}\pi_{R}}{\partial p_{2}\partial s_{1}} \\ \frac{\partial^{2}\pi_{R}}{\partial s_{1}\partial p_{1}} & \frac{\partial^{2}\pi_{R}}{\partial s_{1}\partial p_{2}} & \frac{\partial^{2}\pi_{R}}{\partial{s_{1}}^{2}} \end{matrix} \right]=\left[ \begin{matrix} {2T}_{3} & X_{3} & \gamma_{3} \\ X_{3} & 2\alpha_{3} & \phi_{3} \\ \gamma_{3} & \phi_{3} & 2\lambda_{3} \end{matrix} \right]$$

Since, the second order optimization conditions, can be derived:

$\mathrm{if} {(H}_{11}=\frac{\partial^{2}\pi_{R}}{\partial{p_{1}}^{2}}={2T}_{3}<0), {\det\left( \left[ \begin{matrix} {2T}_{3} & X_{3} \\ X_{3} & 2\alpha_{3} \end{matrix} \right] \right)=4\alpha_{3}T_{3}}-X_{3}^{2}>0 \mathrm{and} \det\left( H \right)=[2T_{3}\left( 4\alpha_{3}\lambda_{3}-\phi_{3}^{2} \right)-X_{3}\left( 2X_{3}\lambda_{3}-\gamma_{3}\phi_{3} \right)+\gamma_{3}\left( X_{3}\phi_{3}-2\alpha_{3}\gamma_{3} \right)]<0 ,$ $\pi_{R} is strictly concave in p_{1}, p_{2}\mathrm{and}s_{1}$.
